# A Computer Vision Approach toward Verifying CFD Models of Stirred Tank Reactors

**DOI:** 10.1021/acs.oprd.4c00229

**Published:** 2024-08-31

**Authors:** Calum Fyfe, Henry Barrington, Charles M. Gordon, Marc Reid

**Affiliations:** † Department of Pure and Applied Chemistry, University of Strathclyde, Glasgow G1 1XL, U.K.; ‡ Scale-up Systems Ltd, 23 Shelbourne Road, Dublin 4 D04 PY68, Ireland

**Keywords:** mixing, computer vision, CFD, kinetics, imaging

## Abstract

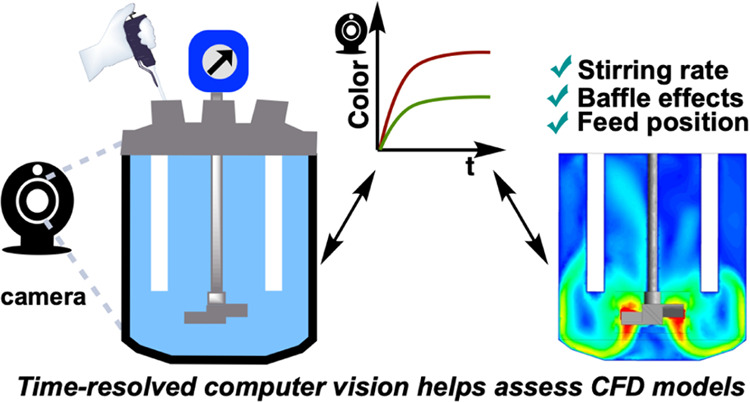

Mixing is one of the most important nonchemical considerations in the design of scalable processes. While noninvasive imaging approaches to deliver a quantifiable understanding of mixing dynamics are well-known, the use of imaging to verify computational fluid dynamics (CFD) models remains in its infancy. Herein, we use colorimetric reactions and our kinetic imaging software, *Kineticolor*, to explore (i) the correlation of imaging kinetics with pH probe measurements, (ii) feed point sensitivity for Villermaux–Dushman-type competing parallel reactions, and (iii) the use of experimental imaging kinetic data to qualitatively assess CFD models. We report further evidence that the influences of the stirring rate, baffle presence, and feed position on mixing in a tank reactor can be informatively captured with a camcorder and help experimentally verify CFD models. Overall, this work advances scarce little precedent in demonstrating the use of computer vision to verify CFD models of fluid flow in tank reactors.

## Introduction

### Mixing and Scale-Up

*“Always assume there is a mixing problem until proven otherwise”* (Dr Ed Paul, AIChE Process Development Symposium, 2003). Mixing phenomena are often neglected during the early research and development stages of new chemical processes, especially in batch. As a result, experimental sections of synthetic methodology publications rarely report on the effects of vessel geometry, stirring rate (except in the case of specific phase-dependent applications),^1−5^ or stirrer bar dimensions.^6^ At the reaction scales typically applied in research laboratories, one might naturally assume that the reactions are under kinetic control, on a scale too small to justifiably consider mechanical (i.e., mixing or mass transfer) influences. It is, again typically but not universally, only on scale-up of a process that mass transfer is considered as a potential limiting factor in the overall process. Having said this, such issues have been considered on the small, high throughput scale.^7^ By-products, attenuated kinetics, and safety concerns might all be emergent on the manufacturing scale where each was undetected or inconsequential on the small scale. Micro, meso and macromixing phenomena become more distinct as a process is scaled up, and any one of the processes may prove to be too difficult to overcome.

Counter to the above points regarding the scale-up of batch processes, the impact of mixer design and channel geometries for small scale applications in flow are well-known to both chemistry and chemical engineering audiences.^8−14^ In such flow chemistry domains, consideration of reaction chemistry *and* engineering more naturally go hand-in-hand during early research and development than is typically the case for processes discovered (then scaled up) in batch.

### Strategies for Mixing Assessment in Tank Reactors

**Figure 1:**
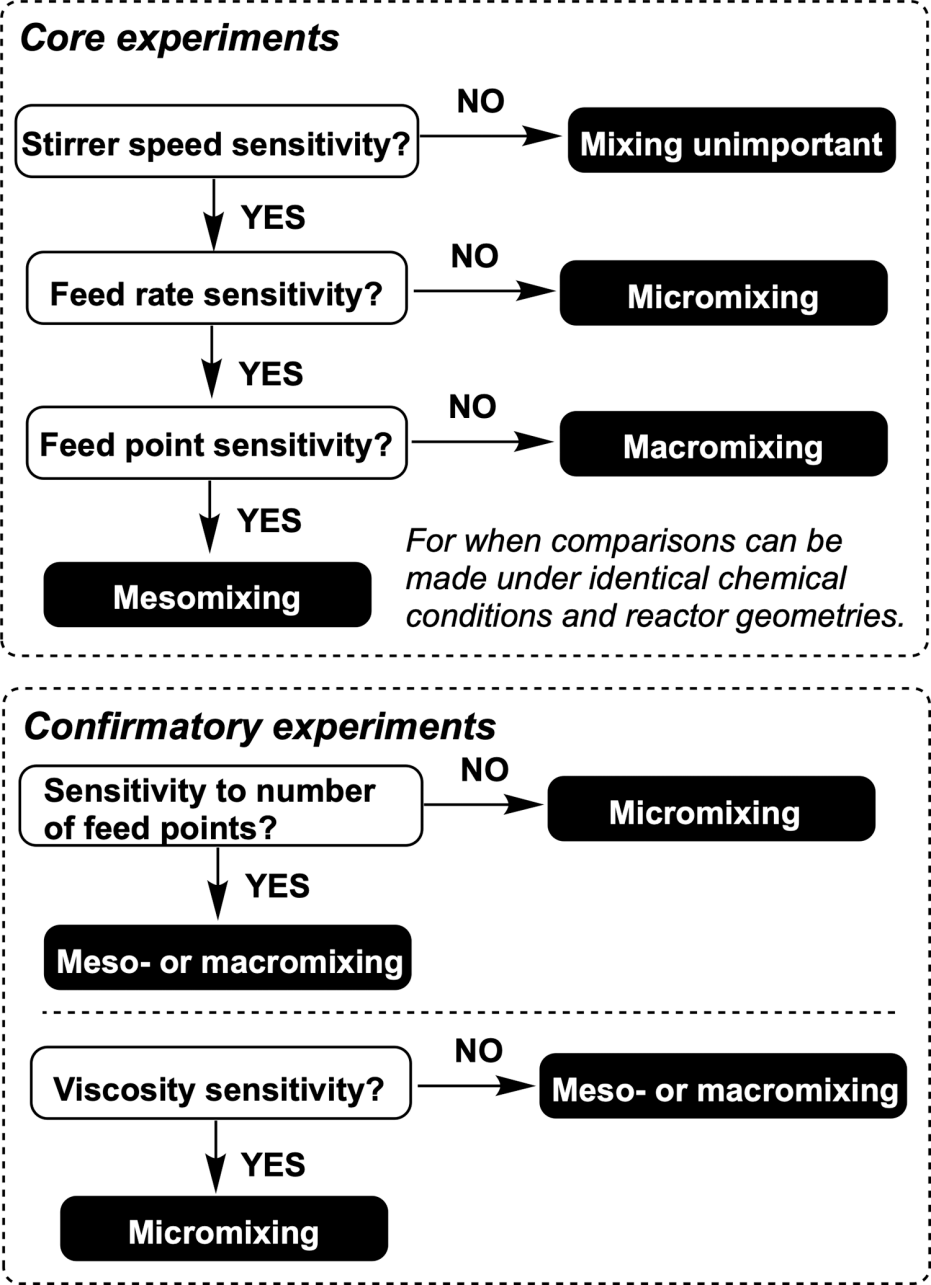
Simplified workflow from the Bourne protocol to establish the different scales of mixing sensitivity (if any) for a reaction.^15^

**Figure 2:**
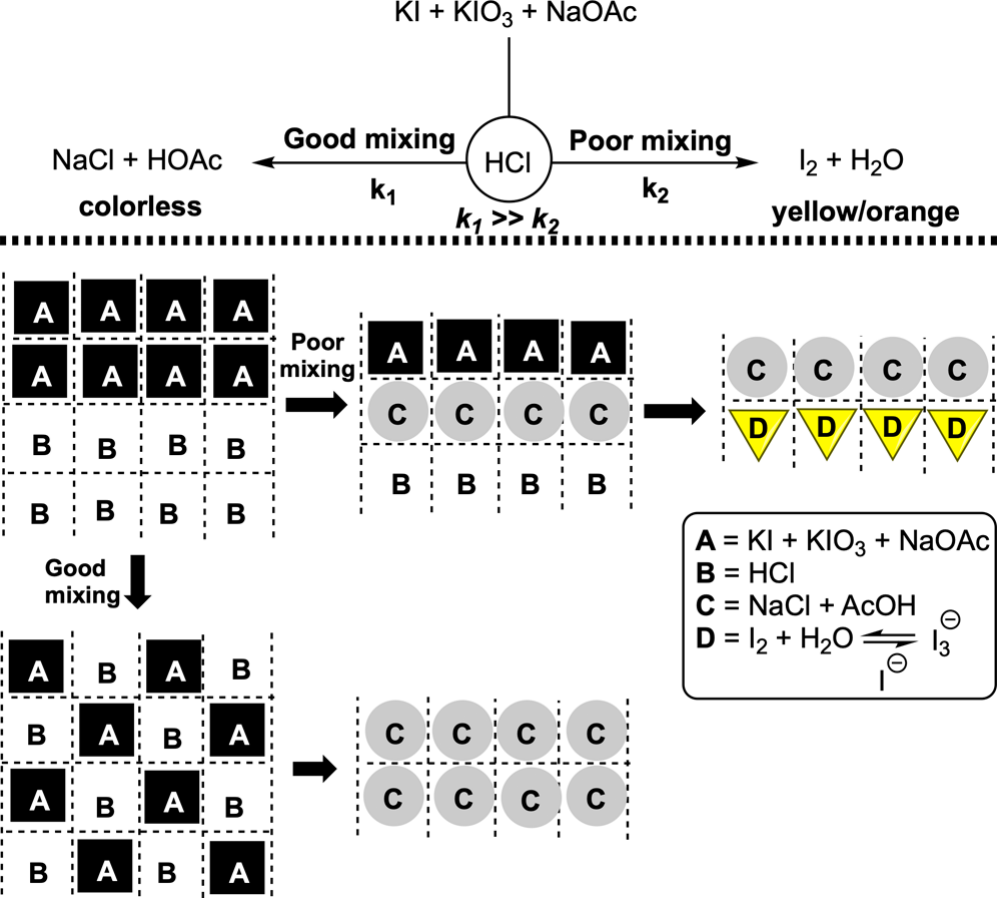
Top: Simplified overview of the modified Villermaux–Dushman reaction. Bottom: The importance of mixing versus reaction time scale for such parallel competitive reactions.

Initially reported in 2003, the Bourne protocol outlines a series of reaction classes that exemplify the value of investigating the impact of impeller speed, feed position, and feed rate on overall mixing control.^15^ The workflow in Figure 1 shows how this protocol can be used to pick apart the influences of micro-, meso-, and macromixing effects in the reaction of interest. The Villermaux–Dushman reaction (and variations thereon) is a visual demonstration of mixing-sensitive parallel competitive neutralization and redox processes. Good mixing favors a fast, colorless acid–base neutralization, and poor mixing (through high local concentration of H^+^) leads to an iodine-forming redox process, visible as a distinct yellow/brown color against the colorless bulk.^16−21^ The redox pathway is typically monitored using spectrophotometry, tracking the subsequent emergence of triiodide (process D in Figure 2). The tell-tale signs of poorer mixing can be inferred from the amount of yellowing that occurs due to the formation of iodine. Building on this precedent, and our last contribution to this journal,^22^ we aimed to broaden the application of such mixing investigations through analysis of video recordings of tank reactors. Ultimately, this work was aimed at providing a meansto experimentally verify computational fluid dynamics (CFD) models.

### Imaging and Computational Fluid Dynamics

CFD employs numerical methods to simulate the behavior of fluid flows. It is a cornerstone of mixing analysis.^23^ By dividing the fluid domain into small computational cells, CFD models can calculate and predict fluid properties like velocity, pressure, and temperature at different points within the domain. Experimentally, camera-enabled, visible-range imaging methods have previously been applied alongside CFD.^24,25^ Colored dyes or tracers have helped qualitatively visualize mixing phenomena during video recording, yet the same techniques have rarely been applied quantitatively by extracting pixel-level information from the images or videos. To better understand the geometric design of staggered ridges in static micromixers, Liu and co-workers used water and aqueous Rhodamine C to qualitatively visualize and verify CFD-calculated Reynolds (*Re*) numbers.^11^

In 2004, Engler and co-workers used CFD to understand stratified, vortex, and engulfment flow regimes in T-shaped micromixers.^10^ Experimental verification came from pressure-derived measurements, but imaging of the mixing of water and rhodamine B-dyed flow channels was used to qualitatively visualize mixing changes in the T-mixer at different flow rates. In the same year, Schönfeld’s team demonstrated the powerful combination of CFD and two complementary colorimetric methods in order to qualitatively and (by a graphical overlay of CFD and photographic images) quantify the representative accuracy of the CFD approach to modeling split-and-recombine micromixers.^9^

Beyond micromixing, several excellent examples of combining CFD with experimental imaging methods come from the groups of Cachon^25^ and Fitschen.^26^ In the former case, grayscale analysis of pixel distribution from recordings of methylene blue tracer mixing was used to experimentally assess various CFD models. This approach helped verify the minimum viable stirring rate required for homogeneous mixing while exploring small scale batch bioreactor designs. In the latter and more recent example, imaging of bromothymol blue pH titrations was used to compare local and global mixing times calculated across a stirred tank reactor fitted with two Rushton turbines spaced along the vertical impeller axis. Jäger’s team later applied the same pH indication method to experimentally track evolution of RGB values in video recordings of rotary disc reactors.^27^ Armenante and co-workers used phenolphthalein’s decoloration reactions to show agreement between experimental and CFD-generated blend times in a United States Pharmacopoeia (USP) compliant vessel.^28^ In a rare but interesting meta-analytical example, Hens’ team employed image analysis to extract insights on velocity distributions using contour plots output from their CFD analyses of chlorination contact tanks.^14^ It is worth acknowledging the related imaging methods of particle image velocimetry (PIV) and planar laser-induced fluorescence (PLIF) which represent powerful methods of capturing flow velocities and concentration fields, respectively.^24,29,30^ However, since laser-based techniques are typically more difficult to safely transfer across scales of operation, these methods are not considered in detail here.

### Kineticolor

**Figure 3:**
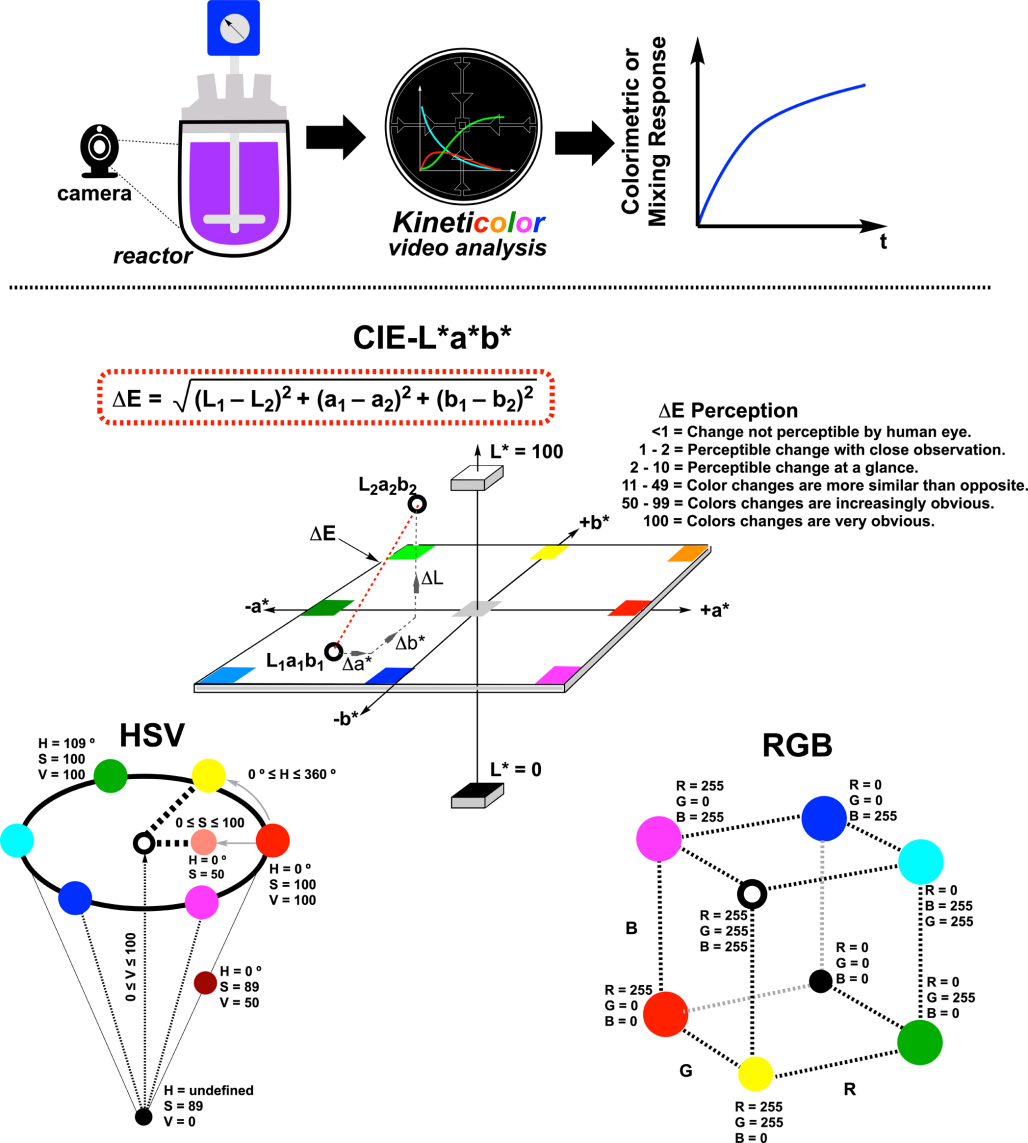
Top: Our computer vision-enabled analysis of chemical reactions, across the scales of development, extracts averaged and spatially resolved (i.e., mixing) metrics from videos of the reaction bulk. Bottom: Visual representations of the key color models used in *Kineticolor*.^35^

Kineticolor is a computer vision software package under ongoing development in our team, and provides a noncontact bulk method for analyzing chemical processes.^22,31−33^ The software uses video input and user-selected regions of interest to track averaged and spatially resolved changes in reaction bulk over time. One such metric that features heavily in this work is the spatially resolved Δ*E* (Figure 3, center), the Euclidean distance between a reference color and another in the CIE–L*a*b* space.^34^ For video analysis, this means that the first frame is the reference against which subsequent frames are compared, producing data that represents the change in Δ*E* over time. Crucially, for this work, such analyses can be globally averaged or, in the case of mixing analysis, spatially segmented into regions across the reactor, as captured on video.^22^

### Aims

Herein, we report our early efforts to assess CFD models using our time-resolved computer vision methods.^36^ To achieve this, we aimed to investigate: (i) the correlation of imaging kinetics with pH probe measurements, (ii) feed point sensitivity for Villermaux–Dushman-type competing parallel reactions, and (iii) which metrics derived from our imaging kinetics approach with *Kineticolor* are most suitable for assessing CFD models experimentally. Building on our earlier computer vision mixing analysis, the current work focuses on tank (or, more broadly, batch) reactors. Hence, we were interested in assessing the potential value of using both experimental imaging and computational fluid dynamics, together, to quantify the impact of stirring rate, baffles, and reagent feed position on reaction and mixing progress.

## Results and Discussion

### CFD Calculations

**Figure 4:**
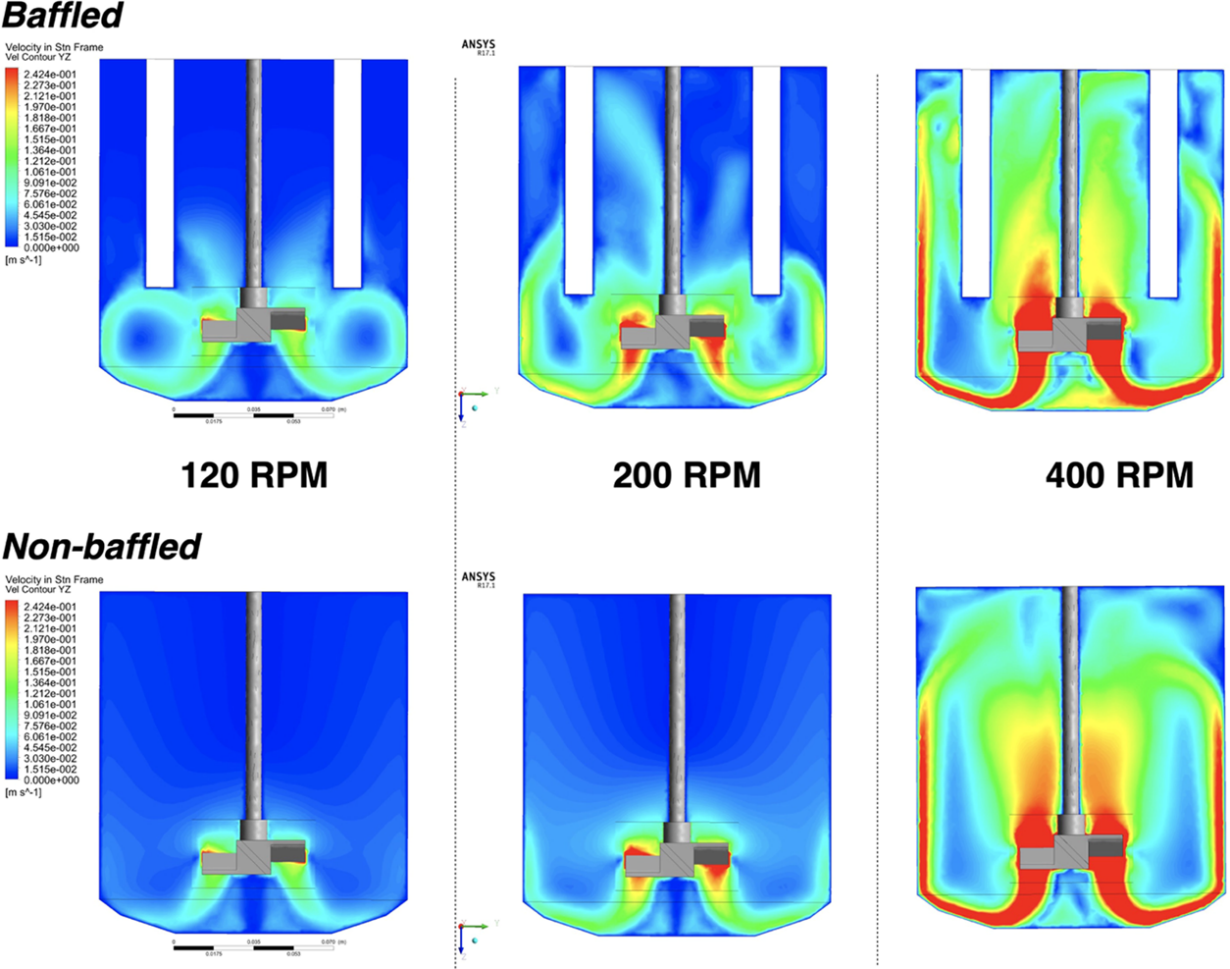
CFD models showing velocity contours for a cross-section at 50% reactor depth at 60 s of a 2L Asynt overhead stirrer tank reactor at various stirring rates, with and without baffle insert, using a four-bladed 45° pitch-blade turbine. Dynamic viscosity, μ = 0.0009 kg m^–1^*s*^–1^.

Alongside stirring rate, it is important to consider the use of baffles when attempting to improve mixing.^37^ The presence of baffles in a vessel promotes top-to-bottom turnover by disrupting flow and introducing vertical (*Z*-axis) vortexes into a system which would otherwise be dominated by the impeller-induced, circular motion on the horizontal XY plane. To simulate stirring and baffle effects, we generated CFD models for our bespoke 2L Asynt overhead stirred tank reactor and pitch-blade turbine impeller, both with and without a beaver tail baffle insert. All subsequent experiments applying computer vision for reaction monitoring were carried out using the same vessel and impeller. Whether baffled or not, it was clear from the cross-sectional diagrams of fluid velocity that increasing stirring rate played a key role in maximizing homogeneity of mixing across the reactor (Figure 4, left to right). Additionally, the same CFD outputs predicted a positive influence of the beaver tail baffles on mixing across the 2L vessel (Figure 4, top row versus bottom row).

The CFD models could also be used to visualize the positive impact of increasing RPM in either the baffled or nonbaffled reactor (Table 1). By plotting the *difference* in fluid velocities derived from the CFD models at each stirring rate, the move from 200 to 400 rpm was predicted to have a greater impact on fluid velocity in the nonbaffled reactor over the baffled variant, especially around the impeller shaft and outer circumference of the reactor. These fluid velocity differences between the nonbaffled and baffled reactors are spatially visualized with largest circles appearing in the key reactor regions of the nonbaffled reactor. The comparatively smaller circles on the velocity difference plot for the baffled reactor showed that moving from 200 to 400 rpm did not change the fluid velocity to the same extent as making the same stirring rate change in the nonbaffled reactor. These data are consistent with a greater influence of radial (i.e., horizontal) fluid flow when baffles are absent.

**Table 1:**
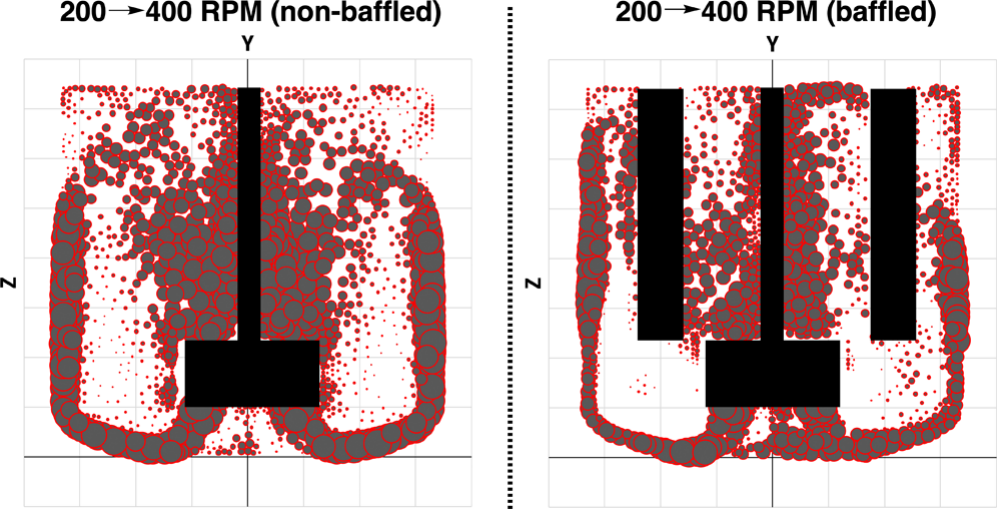
Visualization of and Summarized Metrics from CFD Difference Plots, Showing Where Velocities Increase on Moving from 200 to 400 rpm Stirring Rate^a^

reactor	mean velocity increase	median velocity increase
nonbaffled	8.17	6.33
baffled	5.71	3.77

a Circle diameter maps to the magnitude of positive velocity difference. All values expressed as ×10^–2^ ms^–1^

Qualitatively consistent with the CFD models was a small collection of calculations performed in Dynochem’s Heat and Mass Transfer tool (Table 2). Therein, blend times (a.k.a. macromixing times) were shown to decrease with increasing stirring rate. The same trend was more pronounced with the inclusion of baffles.

**Table 2:** Calculated Power per Unit Mass and Blend Times (a.k.a. Macromixing Times) Simulated Using the Dynochem Mixing and Heat Transfer Tool

stirring rate (RPM)	baffles?	power/mass (W kg^–1^)	blend time (s)	*Re*	turbulent?
120	N	0.001	51.84	3970	N
200	N	0.003	19.72	6620	Y
400	N	0.019	9.81	13,200	Y
120	Y	0.001	31.6	3970	N
200	Y	0.006	14.35	6620	Y
400	Y	0.048	7.18	13,200	Y

With these computed data for our reactor, we next turned our attention to experimental studies on the same reactor to assess our computer vision approach to mixing analysis and its potential to verify CFD models.

### Monitoring Stirring Rate and Baffle Effects with Computer Vision

#### Phenolphthalein Titrations

In our previous mixing-focused contribution to this journal,^22^ we estimated macromixing times (*t*_m_) by visual inspection of apparent plateau regions on the computer vision derived kinetic traces. For the present study, we programmed a more automated approach to plateau detection for quantitative analysis of macromixing effects. These calculations were based on gradient measurements for rolling averages of the color metrics derived from *Kineticolor* analysis of the video footage (see Supporting Information for full details).

**Figure 5:**
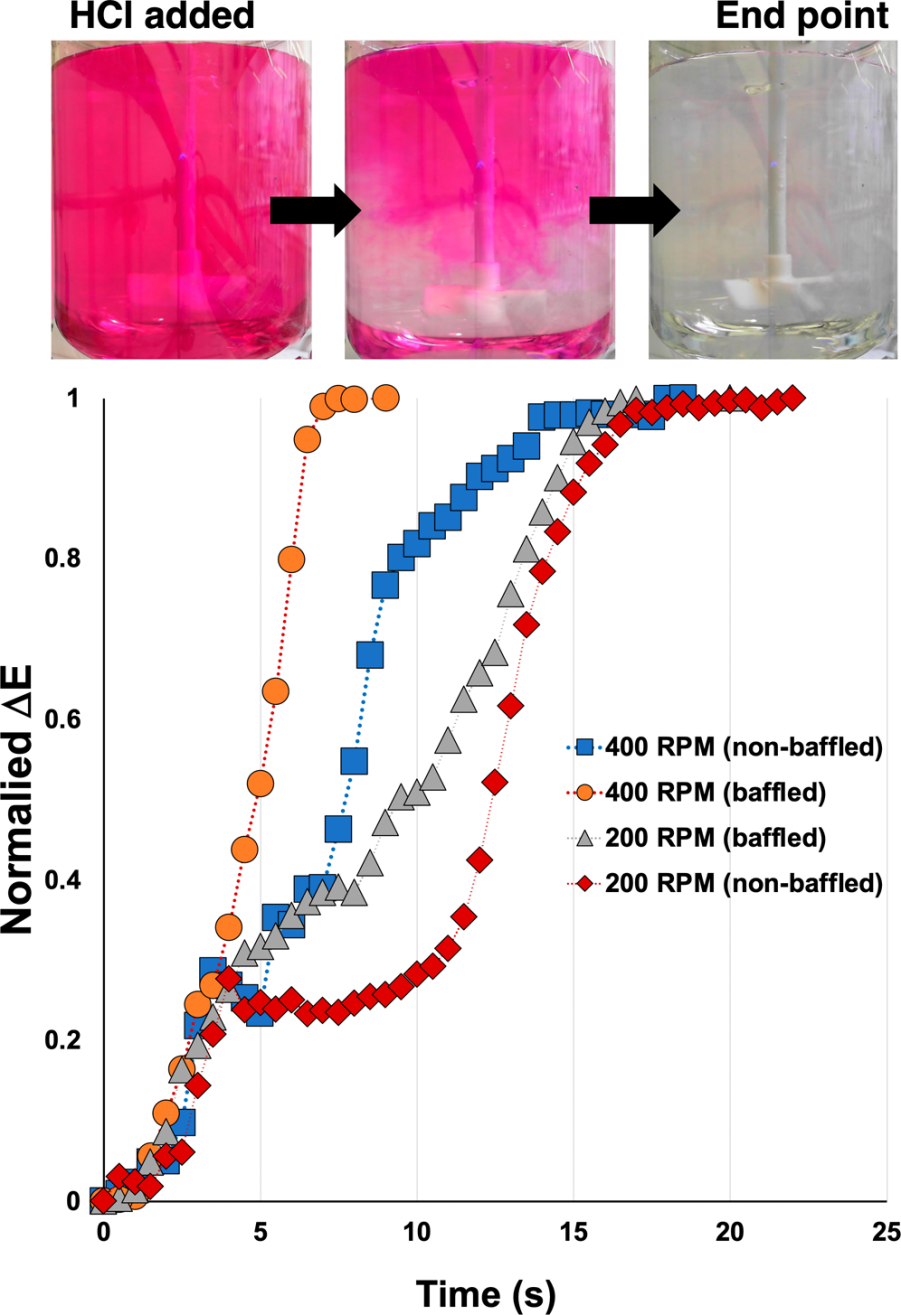
Δ*E* vs time, comparing the impact of baffles at two different stirring rates.

Figure 5 and Table 3 exemplify the use of plateau detection to quantify the sensitivity of titrations of basic phenolphthalein solutions with hydrochloric acid to mixing speed and to the presence or absence of beaver tail baffles. At 200 rpm, our computer vision analysis, averaged across the whole reactor, evidenced little impact of the baffle cage (Figure 5, gray triangles versus red diamonds). However, at 400 rpm, a more distinct positive benefit of a baffled versus nonbaffled tank reactor was captured in the lower *t*_m_ of the baffled over the nonbaffled variant (Figure 5, orange circles versus blue squares).

**Table 3:** Calculated Plateau Times for Time Series Based on Δ*E* Analysis of the Whole Reactor

stirring rate (RPM)	baffles (Y/N)	*t*_m_ (s)
200	N	17.3
200	Y	18.7
400	N	20.0
400	Y	6.5

With reference to the Bourne protocol (Figure 1),^15^ the Δ*E* profiles from Figure 5, averaged over the whole reactor in each case, evidenced a sensitivity to stirring speeds. This observation suggested that at least macromixing effects were playing a role in reaction progress, for the reaction conditions and vessel used. At the same time, the averaged computer vision analysis over the whole reactor cross-section is not granular enough to account for meso-mixing effects, such as the aforementioned vortexing in the nonbaffled reactor. Indeed, the order of *t*_m_ values summarized in Table 3 for the nonbaffled reactor seem, at first, to be at odds with the CFD models, with *t*_m_ at 400 rpm (20.0 s) calculated to be notably larger than that at 200 rpm (17.3 s).

**Figure 6:**
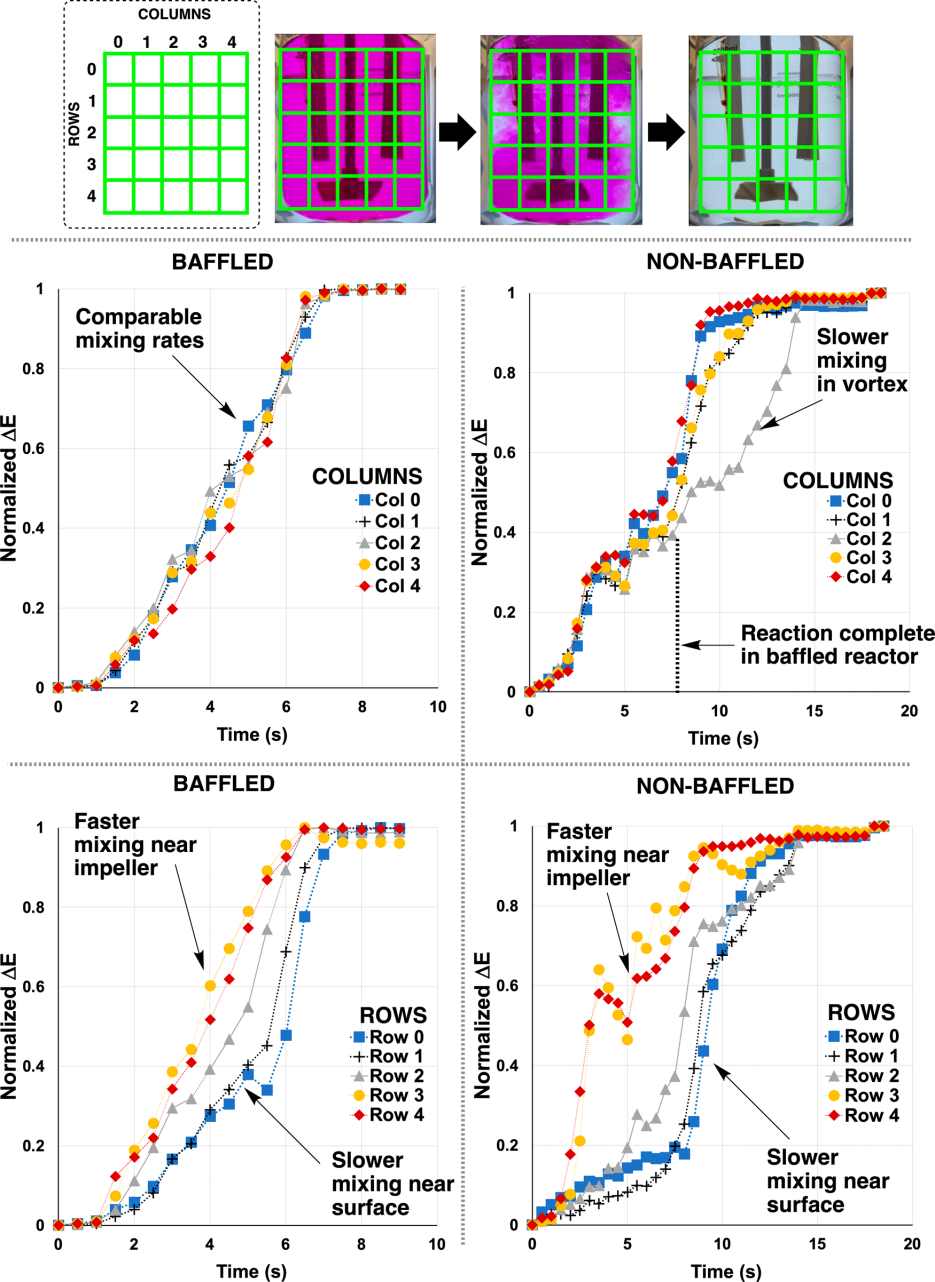
Top: Depiction of grid segmentation of video frame analysis of phenolphthalein titrations. Bottom: Normalized Δ*E* vs time profiles segmented into vertical columns and horizontal rows in the 2D cross-section of reactor, relative to the camera’s field of view. Stirring rate = 200 rpm.

Referring again to the Bourne protocol, further experimentation on HCl feed rates and feed points, beyond stirring rate experiments, would be necessary to investigate meso-mixing effects. However, by segmenting the above Δ*E* profiles from the computer vision analysis into reactor regions, multiple Δ*E* profiles from different parts of the reactor revealed meso-mixing influences on the pH titrations. In Figure 6, the breakdown of Δ*E* into 5 × 5 segments helped reveal meso-mixing effects, without the need for any additional experiments. For the nonbaffled reactor, a vortex around the impeller axis led to elongated mixing time as captured by Δ*E* (see traces labeled “column 2” in Figure 6, top right graph). By comparison, all five Δ*E* columns in the baffled reactor progressed at approximately the same rate (Figure 6, top left graph). The experimentally observed vortex in the nonbaffled reactor was consistent with the zone of high fluid velocity close to the impeller shaft in the 400 rpm CFD model (Figures 4 and 5).

Δ*E* analysis of the baffled and nonbaffled reactors by row, rather than by column, captured additional CFD-consistent evidence of meso-mixing influences (Figure 6, bottom left and bottom right graphs). While more pronounced in the nonbaffled reactor, both reactors showed more rapid rates of color change near the impeller zone of the reactor than near the liquid surface (Figure 6, traces labeled “row 3” and “row 4” versus “row 0” and “row 1”). For all Δ*E* profiles segmented by row and column, the more granular analysis for all 25 cells in the 5 × 5 grid are available in spreadsheets provided as part of the Supporting Information.

The above experiments served to demonstrate the ability to extract spatially resolved information from the video footage, enabling analysis of meso-mixing effects in fewer experiments than outlined in the Bourne protocol.

### pH Meter versus Imaging Insights

**Figure 7:**
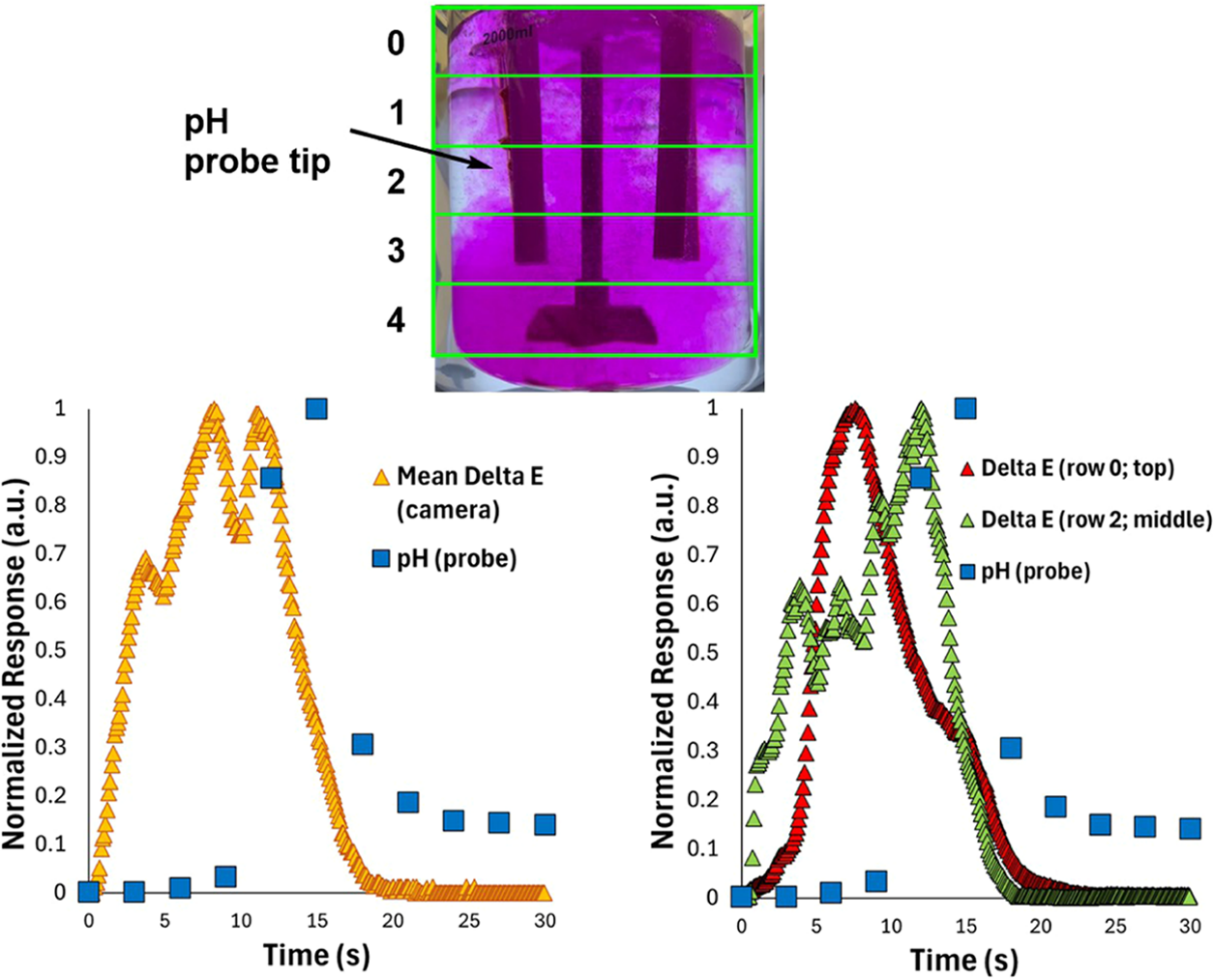
An example of differing response times between pH probe and camera-based measurements of substoichiometric phenolphthalein titration. Top: illustration of row-based segmentation of *Kineticolor* analysis, showing that the pH probe tip sits in row 2. Bottom left: global average Δ*E* and pH measurements versus time. Bottom right: Δ*E* for rows 0 and 2 coplotted with the same pH data.

With regard to independent (noncamera) analysis of the same pH titrations, coanalysis with both video recording and in situ pH probe measurements helped demonstrate the time-resolved benefits of camera-enabled reaction monitoring (Figure 7). When comparing the normalized responses of the pH probe and camera when a substoichiometric quantity of sodium hydroxide was added to an acidic solution of phenolphthalein (causing an emergence and disappearance of the purple coloration associated with pH above 8.3), several important distinctions between the probe- and camera-based reaction monitoring methods became clear:

(i) The temporal resolution (measurements per second) of the camera-enabled computer vision approach is typically much higher than that of the pH probe. Indeed, the extent of this greater data density via the camera is defined by the available frames per second (FPS) on the chosen camera hardware.
(ii) The ability of cameras to capture more data per unit time reveals mixing-related fluctuations in phenolphthalein speciation (over the first 10 s in Figure 7) that do not register on the pH probe in the same time period.
(iii) As recorded by the maximum normalized response of the camera and pH probe, the camera registers the temporary maximum local concentration of hydroxide approximately 8 s before it is recorded by the pH probe (Figure 7, left).
(iv) The dependence on a single point of measurement for pH, based on the probe’s position in the reactor, is demonstrated by the fact that video-derived Δ*E* measured in the row where the pH probe sits gives a closer agreement between the time to maximum response for the camera and the probe (Figure 7, right, where the peak of the green triangles is closer to the peak of the blue squares than is the peak of the red triangles).

#### Villermaux–Dushman Parallel Competing Reactions

**Figure 8:**
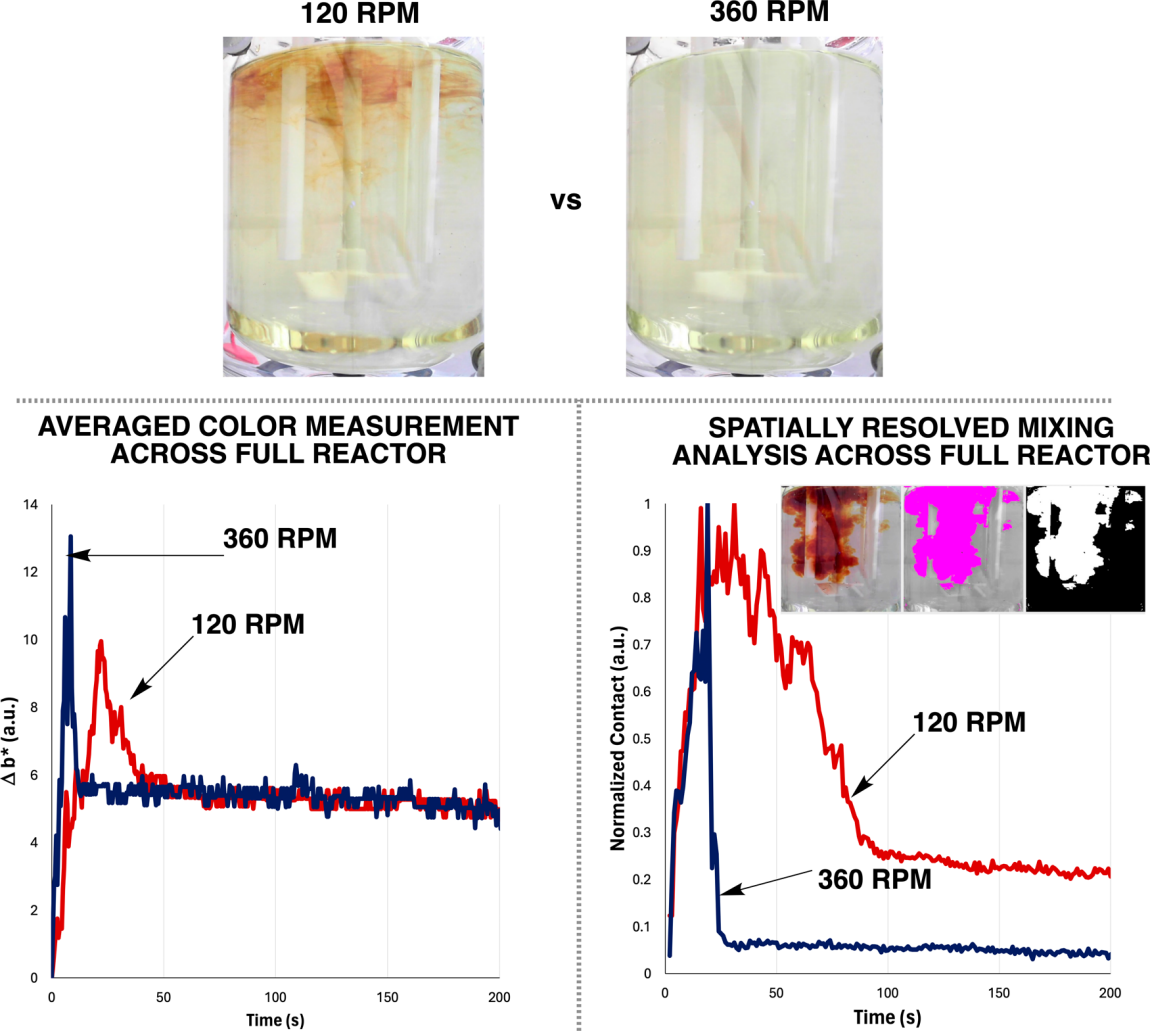
Top: Snapshots of Villermaux–Dushman reaction progress at 30 s after initial HCl charge at the reactor surface. From left to right, increasing stirrer RPM decreases the visually apparent coloration due to iodine formation. Bottom left: A normalized measure of the b* parameter from the CIE–L* a* b* color space maps the average increase in yellowness (positive values) over time. Bottom right: The *Contact* metric is used to measure the effective total perimeter between adjacent light and dark pixels above and below a binary threshold. Whereas the averaged color measurement with b* (left) shows the reactions tracking similar trajectories after 50 s, the spatially resolved *Contact* measurement (right) more clearly draws out the longer-term differences in bulk heterogeneity of the reactions. Bottom right inset: a visualization of *Contact* regions where the left most panel is the normal video, the middle and right show the binary threshold set to capture the relevant coloration spatially.

Applying the Villermaux–Dushman reaction (Figure 2) in place of the phenolphthalein titrations enabled us to explore mixing sensitivities in the same reactor for a more intricate, multistep reaction. Once again guided by Bourne’s protocol, a study on stirring rate revealed clear mixing sensitivities, with lower stirring rates resulting in a more obvious yellow/brown coloration from iodine formation associated with poor mixing (Figure 8, top). Instead of the CIE–L* a* b*-derived contrast metric Δ*E*, we employed the b* component (blue-to-yellow axis of the CIE–L* a* b* color space defined in Figure 3) to capture reaction yellowing over time (Figure 8, bottom left). Focusing on the baffled reactor here, the macromixing times estimated decreased with increased stirring RPM (Table 4). These results were again qualitatively consistent with the CFD models in Figure 4.

**Table 4:** Calculated Plateau Times for Color-Averaged Time Series across the Whole Reactor

stirring rate (RPM)	plateau (s)
40	227.0
120	33.5
360	12.5

**Figure 9:**
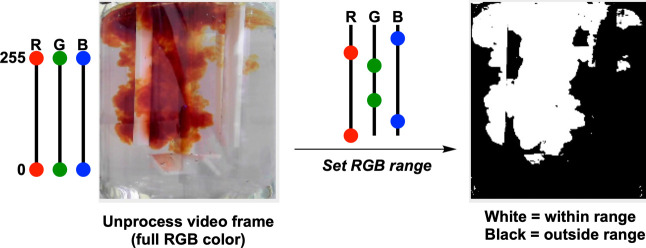
Simplified representation of the color selection method used to obtain a more sensitive RGB threshold versus traditional grayscale to track iodine formation.

For the same Villermaux–Dushman reactions, we again investigated the use of spatially resolved computer vision metrics derived from the video footage to ascertain meso-mixing effects on the reaction, without running any separate experiments on feed point effects. As well as the use of Δ*E* by row or column, we also used this case study to highlight the use of the *Contact* parameter (Figure 8, bottom right).^22^ Here, the reaction coloration was captured by a three-channel (as opposed to grayscale) threshold method, enabling more accurate segmentation of the yellow/brown “plume” visible on addition of acid to the reactor (Figure 9). Using this approach to set image pixels as either black or white, *Contact* then measures the number of pixels around the perimeter in regions where white pixels meet the black pixels. The higher the *Contact* value on the *y*-axis, the larger the total perimeter around white/back pixel boundaries, and the more regions of heterogeneity (incomplete mixing) there are being recorded at a given point in time. In Figure 8 (bottom right), the *Contact* metric is sensitive enough to capture spatially resolved changes happening over an additional 50 s, beyond what was captured using b* via pixel averaging across the entire region of interest analyzed.

#### Mixing Experiments Not Dependent on pH

**Figure 10:**
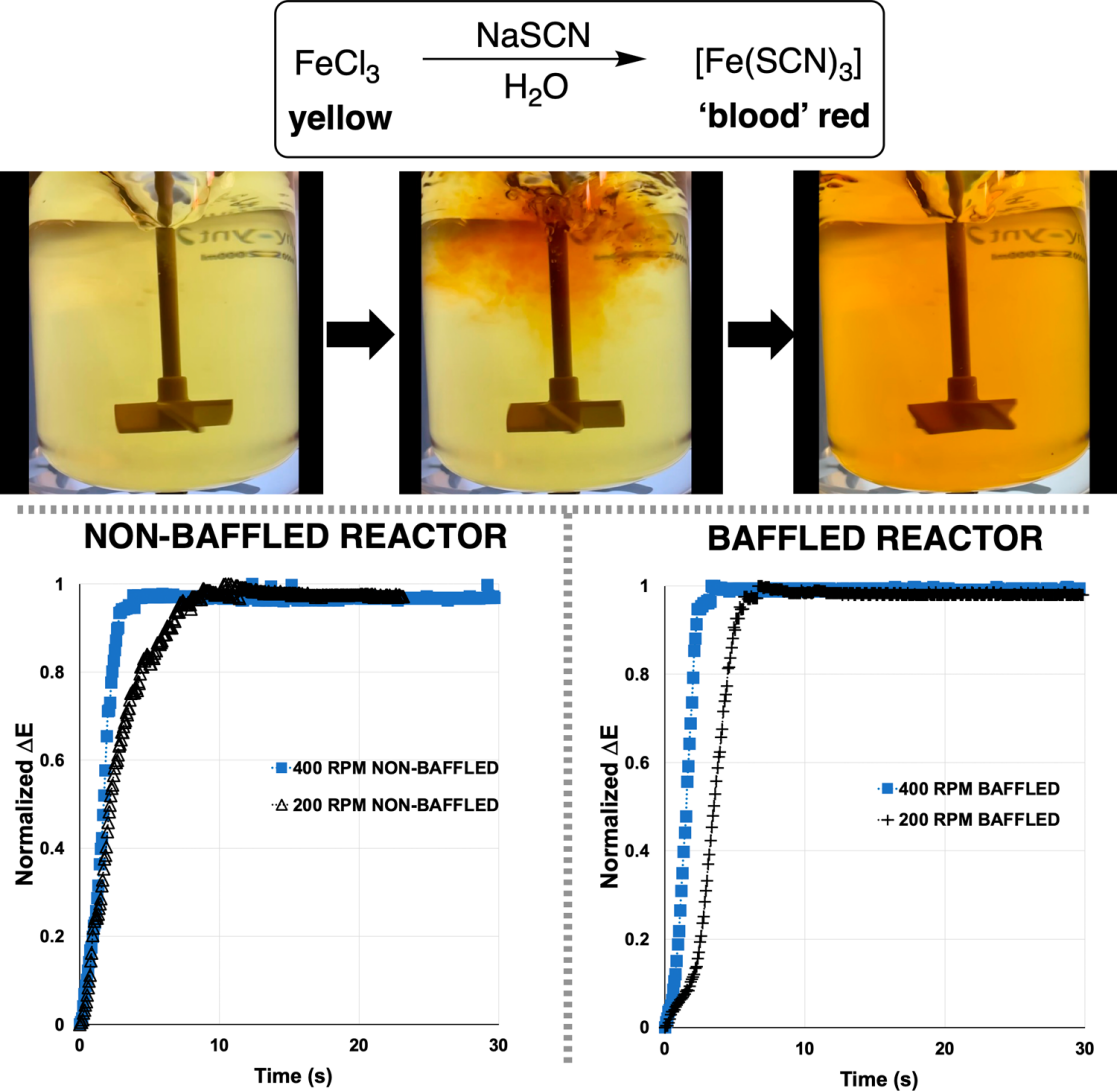
Top: “Reddening” of reaction mixture upon addition of NaSCN to aqueous FeCl_3_. Bottom: representative Δ*E* profiles showing the sensitivity of the reaction to stirring rate.

**Figure 11:**
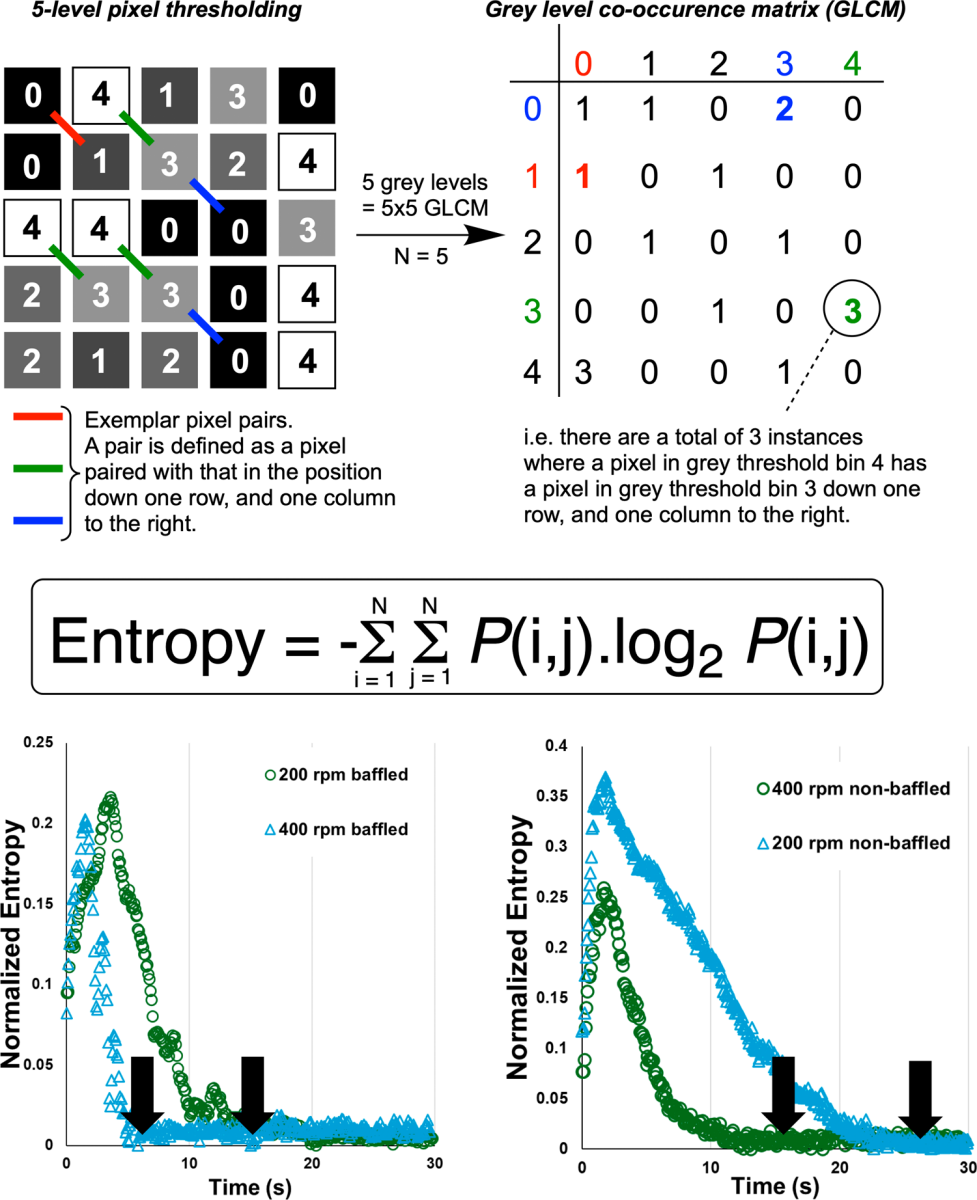
Top: Visual definition of turning greyscale image or video frame into a gray level co-occurrence matrix (GLCM). The resulting GLCM, per frame, can be used to extract metrics like *Entropy*, defined in the boxed equation, where i and j are rows and columns in the GLCM, P is probability, and N is the square dimension of the GLCM related to the number of gray levels employed.^22^ Bottom: Normalized plots of Entropy versus time, as captured from *Kineticolor* analysis of reactor video footage. Higher values of *Entropy* denote more chaotic/random video frames, interpreted as increased heterogeneity. The arrows indicate points where *Entropy* plateaus, below a set threshold, denoting macromixing times.

The stirring rate influences of our reactor could also be assessed using systems not dependent on pH or Brønsted acid–base chemistry. To this end, we employed thiocyanate substitution at Fe(III), known among mixing experts to be useful on account of the high contrast color change,^8^ (Figure 10, Table 5). The computer vision analysis revealed that, like the acid–base chemistry, the iron chemistry, in the same 2L vessel, was sensitive to stirring rate and baffles. Notably, when using a more spatially sensitive texture-derived metric like *Entropy* (higher values denote a more disordered image), changes could be detected over a longer time period than using averaged color. As a result, a clearer distinction of resultant plateau times associated with macromixing could be extracted (Figure 11, bottom).

**Table 5:** Calculated Plateau Times for Time Series Based on Δ*E* Analysis of the Whole Reactor for the Coordination Chemistry Employed in Figures 10 and 11

stirring rate (RPM)	baffles (Y/N)	plateau via Δ*E*(s)	plateau via entropy (s)
200	N	9.00	23.3
200	Y	7.08	13.0
400	N	4.33	12.2
400	Y	3.66	4.8

**Figure 12:**
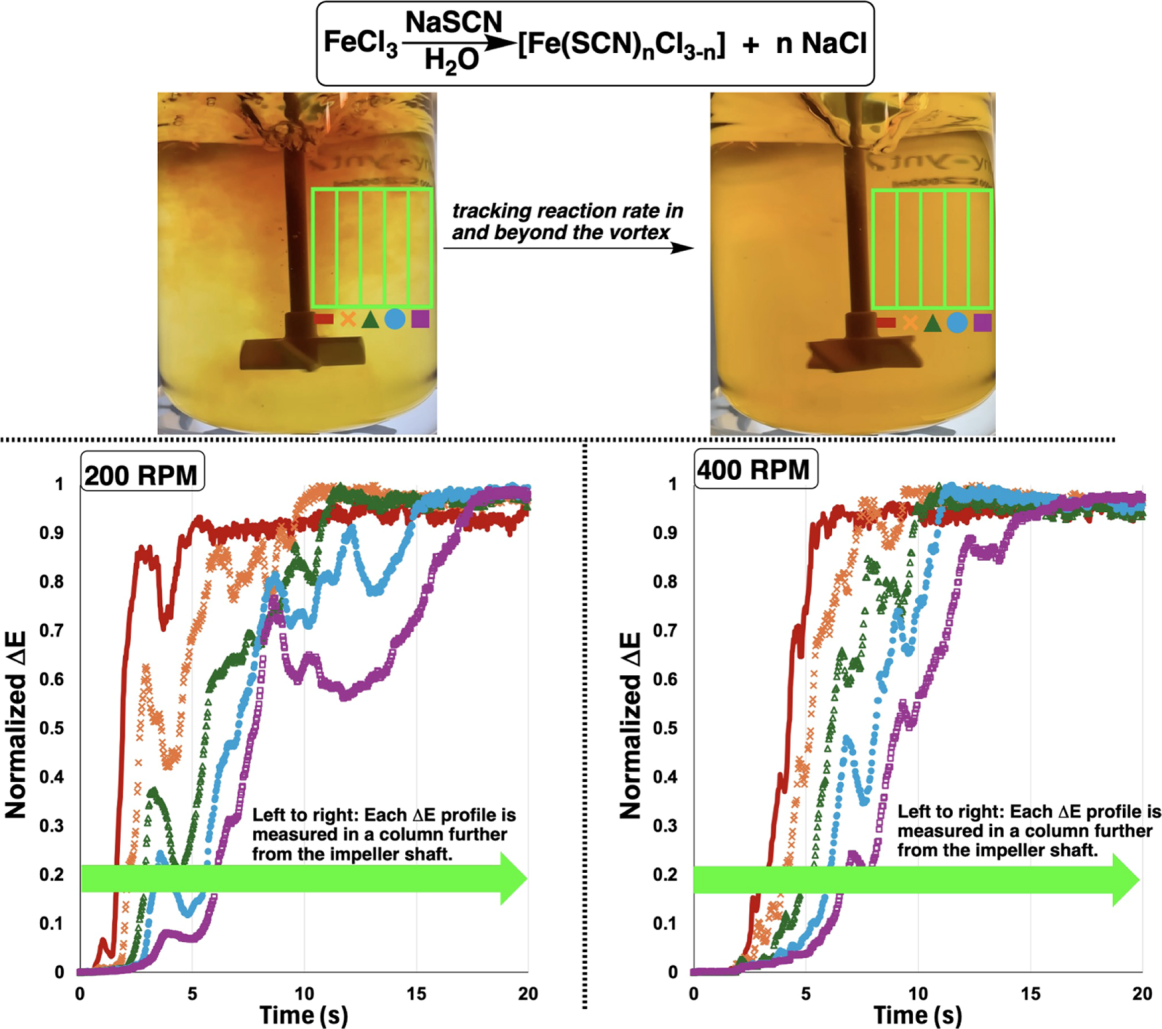
Top: Stills from a video of substitution of chloride with thiocyanate at Fe(III), with dark brown region on the left-hand image evidencing a vortex. The green overlay depicts the region of interest in the video analyzed by *Kineticolor*. Bottom: Δ*E* profiles for each of the five columns in the region of interest.

The same iron coordination chemistry was useful in further analyzing the vortexes in the nonbaffled reactor (see earlier CFD discussion, Figures 4 and 5). In order to track the formation of iron thiocyanate derivatives that cause a darkening of the reaction mixture, we set up a region of interest to be analyzed by vertical column, starting from the impeller shaft and moving outward (Figure 12). Using the Δ*E* contrast metric, it was possible to show that the vortex in the nonbaffled reactor, at both 200 and 400 rpm, resulted in attenuated rates of reaction further from the impeller shaft. Related vortex analyses for the phenolphthalein titrations are available in the Supporting Information.

Together, the experiments on stirring rate, analyzed by computer vision (in this case, via *Kineticolor*), had enabled some qualitative assessment of the CFD models constructed for the same reactor. Maintaining guidance from the Bourne protocol, we next sought to extend the computer vision analysis to experiments in which other parameters beyond stirring rate and baffle presence were varied.

### Feed Point Sensitivity

While our investigation showed that the spatially resolved video analysis could help reveal meso-mixing sensitivities without the need for additional feed point experiments, we nonetheless explored feed point effects to check for consistency in our interpretation of the *Kineticolor*-derived data sets and, indeed, the initial CFD models.

**Figure 13:**
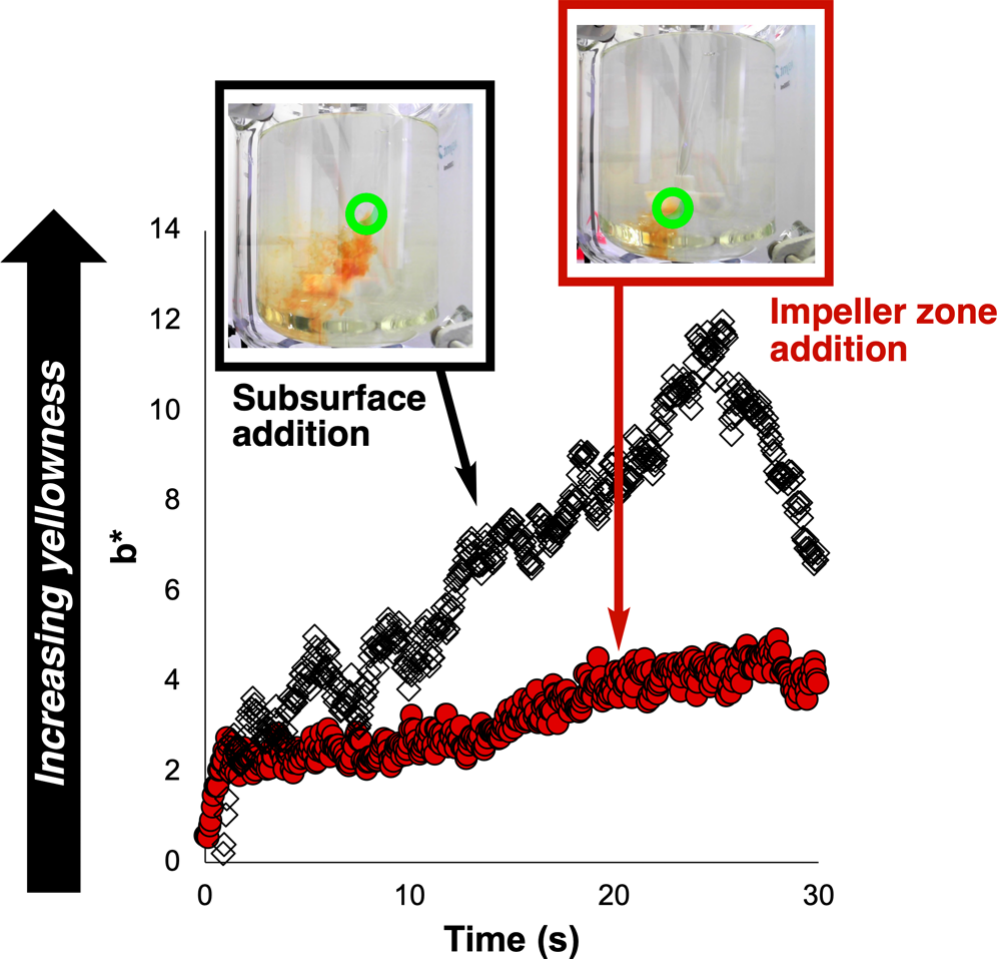
b* profiles of Villermaux–Dushman reactions to investigate the impact of HCl feed position in 2L tank reactor. HCl injection from the subsurface zone led to detection of higher yellow coloration than injection in the impeller zone. More positive b* values infer more yellowing at a given point in time relative to lower (more negative) b* values. Green circles shown on the inset images show the subsurface and impeller zone feed points. Stirring rate = 120 rpm.

**Figure 14:**
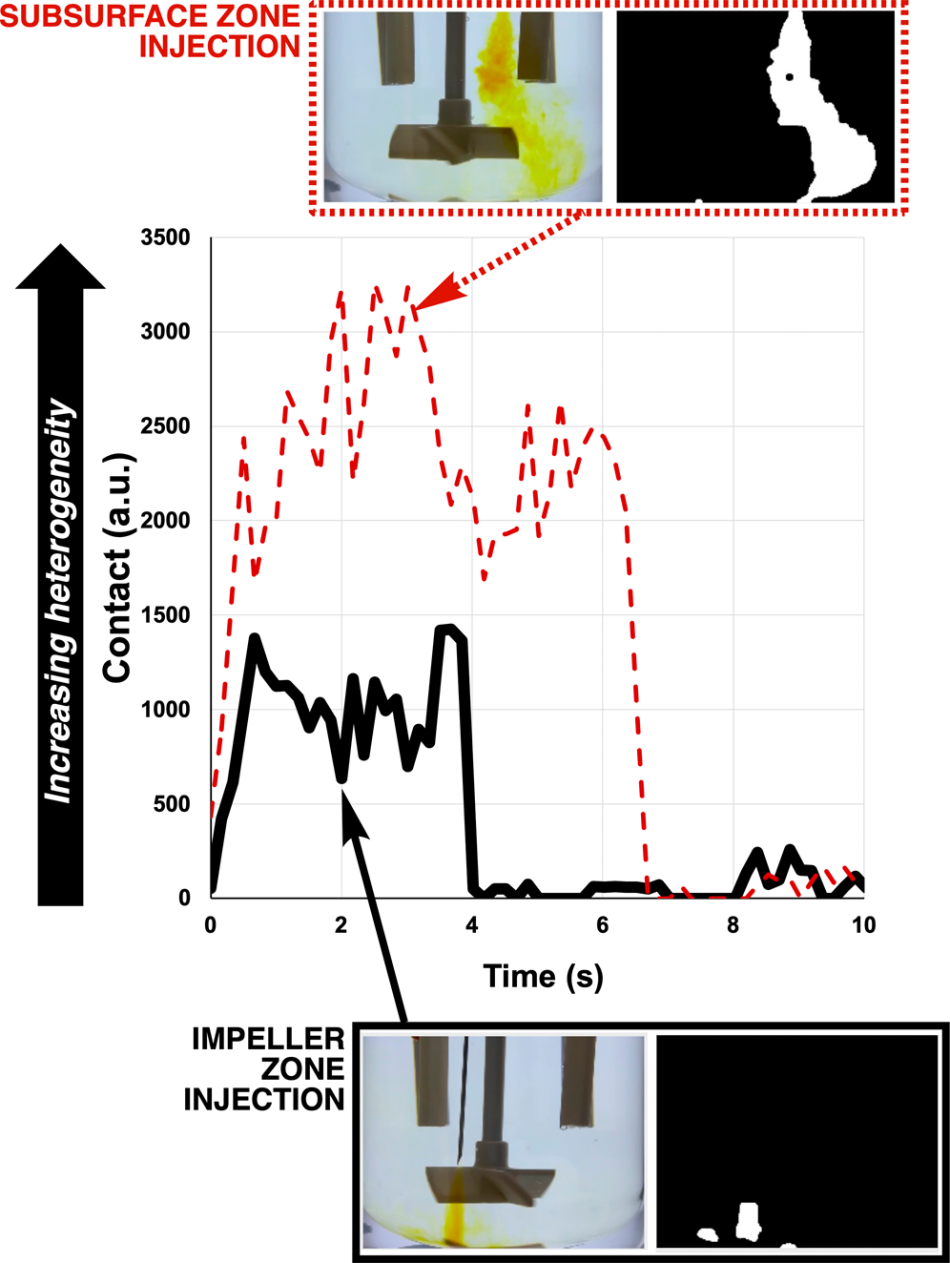
Spatially resolved profiles of reaction mixture heterogeneity using the *Contact* metric. In each image, the raw video footage is shown on the left. On the right, the three-channel threshold images highlight the areas of yellow coloration detected. The total perimeter around the boundary of white and black regions defines the contact magnitude. Stirring rate = 120 rpm.

Focusing first on the Villermaux–Dushman chemistry, the impact of feed position on mixing in our 2L tank reactor was visibly apparent on account of the differing amounts of iodine formation for HCl addition at two different feed points. These effects were also captured in *Kineticolor* analysis of the video footage (Figure 13). With the b* parameter of the CIE–L* a* b* color space, the more positive the value the more yellow the solution appears to be. The b* value is most positive for addition of acid further from the impeller. Impeller zone addition, on the other hand, had a markedly lower maximum observed b* value, indicating more effective mixing. This assessment was consistent with the related Villermaux–Dushman experiments set up to enable spatially resolved *Contact* calculations to reveal the dependency of reaction yellowing on feed position (Figure 14).

Once again referring to the initial CFD models, all cases predicted maximum velocities around the impeller itself, decreasing vertically as one moves toward the liquid surface.

## Conclusions

Utilizing noncontact, camera-enabled computer vision analysis of a tank reactor has enabled the development of methods toward the experimental verification of CFD models of the same reactor. The impact of stirring rate, baffles, and feed point have been investigated, providing a quantified means by which to compare mixing efficiencies under different circumstances, and thus assess whether or not the CFD models can predict the same effect. Practically useful outputs from the study include an experimental measure of vortex effects on mixing for nonbaffled reactors, and an assessment of response time differences between spatially resolved computer vision measurements and pH measurements taken by a probe, located at a single point in the reactor.

Future work will require additional assessment of feed rate as well as feed position, as this represents a limitation of the current study. Additionally, efforts to derive direct, fully quantitative assessment of CFD accuracy from ground truth imaging data are ongoing in our laboratory.

## Data Availability

In addition to the higher level details shared in the Supporting Information PDF document, a zipped folder of machine-readable data, ordered according to the figure and table numbers in the main text, is available on figshare at 10.6084/m9.figshare.26405800.v2

## Supporting Information Available

Experimental procedures, reactor setup, CFD calculations, and computer vision data in machine-readable formats wherever possible (PDF)

## References

[ref1] Luo J.; Wu Y.; Zijlstra H. S.; Harrington D. A.; McIndoe J. S. Mass transfer and convection effects in small-scale catalytic hydrogenation. Catal. Sci. Technol. 2017, 7, 2609–2615. 10.1039/C7CY00492C.

[ref2] Kano T.; Aota Y.; Maruoka K. Rate Acceleration of Solid-Liquid Phase-Transfer Catalysis by Rotor-Stator Homogenizer. Adv. Synth. Catal. 2016, 358, 2996–2999. 10.1002/adsc.201600425.

[ref3] Denmark S. E.; Weintraub R. C.; Gould N. D. Effects of Charge Separation, Effective Concentration, and Aggregate Formation on the Phase Transfer Catalyzed Alkylation of Phenol. J. Am. Chem. Soc. 2012, 134, 13415–13429. 10.1021/ja304808u. 22856542

[ref4] Lennox A. J. J.; Lloyd-Jones G. C. Organotrifluoroborate Hydrolysis: Boronic Acid Release Mechanism and an Acid-Base Paradox in Cross-Coupling. J. Am. Chem. Soc. 2012, 134, 7431–7441. 10.1021/ja300236k. 22512340

[ref5] Beutner G. L.; Coombs J. R.; Green R. A.; Inankur B.; Lin D.; Qiu J.; Roberts F.; Simmons E. M.; Wisniewski S. R. , Palladium-Catalyzed Amidation and Amination of (Hetero)aryl Chlorides under Homogeneous Conditions Enabled by a Soluble DBU/NaTFA Dual-Base System. Org. Process Res. Dev. 2019, 23, 1529–1537. 10.1021/acs.oprd.9b00196.

[ref6] Thavarajah R.; Penny M. R.; Torii R.; Hilton S. T. Rapid Lewis Acid Screening and Reaction Optimization Using 3D-Printed Catalyst-Impregnated Stirrer Devices in the Synthesis of Heterocycles. J. Org. Chem. 2023, 88, 16845–16853. 10.1021/acs.joc.3c01601. 38011901 PMC10729026

[ref7] Townley C.; Branduardi D.; Chessari G.; Cons B. D.; Griffiths-Jones C.; Hall R. J.; Johnson C. N.; Ochi Y.; Whibley S.; Grainger R. Enabling synthesis in fragment-based drug discovery (FBDD): microscale high-throughput optimization of the medicinal chemist’s toolbox reactions. RSC Med. Chem. 2023, 14, 2699–2713. 10.1039/D3MD00495C. 38107176 PMC10718589

[ref8] Hessel V.; Hardt S.; Löwe H.; Schönfeld F. Laminar mixing in different interdigital micromixers: I. Experimental characterization. AIChE J. 2003, 49, 566–577. 10.1002/aic.690490304.

[ref9] Schönfeld F.; Hessel V.; Hofmann C. An optimized split-and-recombine micro-mixer with uniform chaotic’ mixing. Lab Chip 2004, 4, 65–69. 10.1039/B310802C. 15007443

[ref10] Engler M.; Kockmann N.; Kiefer T.; Woias P. Numerical and experimental investigations on liquid mixing in static micromixers. Chem. Eng. J. 2004, 101, 315–322. 10.1016/j.cej.2003.10.017.

[ref11] Fu X.; Liu S.; Ruan X.; Yang H. Research on staggered oriented ridges static micromixers. Sens. Actuators, B 2006, 114, 618–624. 10.1016/j.snb.2005.06.023.

[ref12] Trad Z.; Fontaine J.-P.; Larroche C.; Vial C. Experimental and numerical investigation of hydrodynamics and mixing in a dual-impeller mechanically-stirred digester. Chem. Eng. J. 2017, 329, 142–155. 10.1016/j.cej.2017.07.038.

[ref13] Kihara T.; Obata H.; Hirano H. Quantitative visualization of fluid mixing in slug flow for arbitrary wall-shaped microchannel using Shannon entropy. Chem. Eng. Sci. 2019, 200, 225–235. 10.1016/j.ces.2019.02.007.

[ref14] Gun S.; Chatterjee S.; Hens A. CFD based analysis of chlorination contact tank design. Mater. Today: Proc. 2022, 57, 1813–1818. 10.1016/j.matpr.2021.12.533.

[ref15] Bourne J. R. Mixing and the selectivity of chemical reactions. Org. Process Res. Dev. 2003, 7, 471–508. 10.1021/op020074q.

[ref16] Dushman S. The rate of the reaction between iodic and hydriodic acids. J. Phys. Chem. A 1904, 8, 453–481. 10.1021/j150061a001.

[ref17] Fournier M. C.; Falk L.; Villermaux J. A new parallel competing reaction system for assessing micromixing efficiency - Experimental approach. Chem. Eng. Sci. 1996, 51, 5053–5064. 10.1016/0009-2509(96)00270-9.

[ref18] Guichardon P.; Falk L. Characterisation of micromixing efficiency by the iodideiodate reaction system. Part I: experimental procedure. Chem. Eng. Sci. 2000, 55, 4233–4243. 10.1016/S0009-2509(00)00068-3.

[ref19] Commenge J. M.; Falk L. Villermaux-Dushman protocol for experimental characterization of micromixers. Chem. Eng. Process.: Process Intensif. 2011, 50, 979–990. 10.1016/j.cep.2011.06.006.

[ref20] Pinot J.; Commenge J. M.; Portha J. F.; Falk L. New protocol of the Villermaux-Dushman reaction system to characterize micromixing effect in viscous media. Chem. Eng. Sci. 2014, 118, 94–101. 10.1016/j.ces.2014.07.010.

[ref21] Guichardon P.; Baqueiro C.; Ibaseta N. Villermaux-Dushman Test of Micromixing Characterization Revisited: Kinetic Effects of Acid Choice and Ionic Strength. Ind. Eng. Chem. Res. 2021, 60, 18268–18282. 10.1021/acs.iecr.1c03208.

[ref22] Barrington H.; Dickinson A.; McGuire J.; Yan C.; Reid M. Computer Vision for Kinetic Analysis of Lab- and Process-Scale Mixing Phenomena. Org. Process Res. Dev. 2022, 26, 3073–3088. 10.1021/acs.oprd.2c00216. 36437899 PMC9680030

[ref23] Fletcher D. F. The future of computational fluid dynamics (CFD) simulation in the chemical process industries. Chem. Eng. Res. Des. 2022, 187, 299–305. 10.1016/j.cherd.2022.09.021.

[ref24] de Lamotte A.; Delafosse A.; Calvo S.; Delvigne F.; Toye D. Investigating the effects of hydrodynamics and mixing on mass transfer through the free-surface in stirred tank bioreactors. Chem. Eng. Sci. 2017, 172, 125–142. 10.1016/j.ces.2017.06.028.

[ref25] Allonneau C.; Olmos E.; Guyot S.; Ferret E.; Gervais P.; Cachon R. Hydrodynamic characterization of a new small-scale reactor mixed by a magnetic bar. Biochem. Eng. J. 2015, 96, 29–37. 10.1016/j.bej.2014.12.005.

[ref26] Fitschen J.; Hofmann S.; Wutz J.; Kameke A. V.; Hoffmann M.; Wucherpfennig T.; Schlüter M. Novel evaluation method to determine the local mixing time distribution in stirred tank reactors. Chem. Eng. Sci.: X 2021, 10, 100098 10.1016/j.cesx.2021.100098.

[ref27] Jäger L.; Scholl S. Experimental characterization and mixing modeling of a horizontally rotating disc reactor. Chem. Eng. Sci. 2023, 280, 118995 10.1016/j.ces.2023.118995.

[ref28] Pace J.; Sirasitthichoke C.; Armenante P. M. Experimental determination and computational prediction of blend time in the USP dissolution testing Apparatus 1. Chem. Eng. Res. Des. 2023, 194, 705–721. 10.1016/j.cherd.2023.05.008.

[ref29] Litvinov I.; Yoon J.; Noren C.; Stöhr M.; Boxx I.; Geigle K. P. Time-resolved study of mixing and reaction in an aero-engine model combustor at increased pressure. Combust. Flame 2021, 231, 111474 10.1016/j.combustflame.2021.111474.

[ref30] Galindo J.; Navarro R.; Tarí D.; Moya F. Analysis of condensation and secondary flows at three-way junctions using optical visualization techniques and computational fluid dynamics. Int. J. Multiphase Flow 2021, 141, 103674 10.1016/j.ijmultiphaseflow.2021.103674.

[ref31] Yan C.; Fyfe C.; Minty L.; Barrington H.; Jamieson C.; Reid M. Computer vision as a new paradigm for monitoring of solution and solid phase peptide synthesis. Chem. Sci. 2023, 14, 11872–11880. 10.1039/D3SC01383A. 37920332 PMC10619640

[ref32] Bugeja N.; Oliver C.; McGrath N.; McGuire J.; Yan C.; Carlysle-Davies F.; Reid M. Teaching old presumptive tests new digital tricks with computer vision for forensic applications. Digital Discovery 2023, 2, 1143–1151. 10.1039/D3DD00066D. 38013815 PMC10408571

[ref33] Yan C.; Cowie M.; Howcutt C.; Wheelhouse K. M. P.; Hodnett N. S.; Kollie M.; Gildea M.; Goodfellow M. H.; Reid M. Computer vision for non-contact monitoring of catalyst degradation and product formation kinetics. Chem. Sci. 2023, 14, 5323–5331. 10.1039/D2SC05702F. 37234891 PMC10208035

[ref34] Mokrzycki W. S.; Tatol M. Color difference E: a survey. Mach. Graphics Vision 2011, 20 (4), 383–411.

[ref35] Capitán-Vallvey L. F.; López-Ruiz N.; Martínez-Olmos A.; Erenas M. M.; Palma A. J. Recent developments in computer vision-based analytical chemistry: A tutorial review. Anal. Chim. Acta 2015, 899, 23–56. 10.1016/j.aca.2015.10.009. 26547492

[ref36] Fyfe C.; Barrington G. C. M.; Henry; Reid M. A Computer Vision Approach Towards Verifying CFD Models of Stirred Tank Reactors. ChemRxiv, 2024. 10.1021/acs.oprd.4c00229 PMC11421076 39323895

[ref37] Nere N. K.; Patwardhan A. W.; Joshi J. B. Liquid-Phase Mixing in Stirred Vessels: Turbulent Flow Regime. Ind. Eng. Chem. Res. 2003, 42, 2661–2698. 10.1021/ie0206397.

