# Supplementary material for: A Computer Vision Approach toward Verifying CFD Models of Stirred Tank Reactors
Source: Org Process Res Dev. 2024 Aug 31;28(9):3661–73. doi: 10.1021/acs.oprd.4c00229 (PMC11421076; doi:10.1021/acs.oprd.4c00229)
Supplement: Supplementary file 1 — op4c00229_si_001.pdf [file op4c00229_si_001.pdf]

A Computer Vision Approach Towards Verifying CFD Models of Stirred Tank Reactors

Calum Fyfe,† Henry Barrington,† Charles M. Gordon,‡ and Marc Reid∗,†

†Department of Pure and Applied Chemistry, University of Strathclyde, Glasgow G1 1XL, U.K.
‡Scale-up Systems Ltd, 23 Shelbourne Road, Dublin 4, D04 PY68, Ireland.

Supporting Information

Table of Contents

Experimental Details *2*
Reactor Setup 2
Phenolphthalein Indicated Neutralisations (Figures 5 –7) 2
Villermaux-Dushman Reaction (Figure 8, 13 and 14) 2
Iron Chloride Displacement Reactions (Figure 10 – 12) 3
Computational Details *4*
Figure 4 - CFD models 4
Computational Fluid Dynamics Modelling with Ansys R17.1 4
Table 1 - CFD Difference Maps 11
Table 2 - DynoChem Calculations 12
Plateau Analysis 15
General Method 15

NOTE – in addition to the higher-level details shared in this document, a zipped folder of machine-readable data, ordered according to the Figure and Table numbers in the main text, is available as part of the supporting information via figshare:

https://figshare.com/articles/dataset/Supporting_Information_zip/26405800

For information on licensing Kineticolor software, please contact the corresponding author and the University of Strathclyde technology transfer office:

marc.reid.100@strath.ac.uk; iprmanager@strath.ac.uk

Experimental Details

Reactor Setup

All experiments were conducted in a two-litre jacketed Asynt reactor with a four-pitch blade stirrer employed at a height of 2.5 cm above the bottom of the reactor. A variable speed overhead stirrer (Velp Scientifica, OHS 60 Digital) was employed. Baffled experiments used a four flat plate radially spaced at 90 degrees baffle insert.

Phenolphthalein Indicated Neutralisations (Figures 5 –7)

1.8 L of water followed by 200 mL 0.1 M NaOH (aq.) and 5 mL of phenolphthalein indicator solution was pipetted into the reactor.

Mixing was commenced for to ensure homogeneous solution. Once a uniform fuchsia/pink solution had developed, recording was started, using a Microsoft LifeCam Studio webcam and Windows 11 camera app.

20 mL of 1 M HCl was then added to the reactor in one rapid charge from a beaker to minimise the influence of addition rate.

For **Figure 5** experiments were conducted at 200 RPM and 400 RPM, baffled and unbaffled.

For **Figure 6 and 7** experiments were conducted at 200 RPM baffled and unbaffled, acid was added as fast as possible by squeezing the pipette bulb. The radial position of the HCl addition was approximately half the vessel depth from the vessel wall inward towards the impeller shaft.

A Thermo Scientific Orion Star A100 Series Benchtop pH/mV/Temperature Meter was used to record the pH. The pH probe was secured to the reactor lid, due to the limited length of the probe, the probe could only be used to measure the pH close to the surface. pH recording was commenced at the same time as video recording.

Villermaux-Dushman Reaction (Figure 8, 13 and 14)

A 200 mL aqueous solution of potassium iodide (5.312 g), potassium iodate (1.284 g), and sodium hydroxide (3.6 g) was prepared and transferred to the reactor and made up with 1.8L water. Stirring (RPM) was started and left for at least a minute to ensure flow had reached a steady state. Recording started and an aqueous hydrochloric acid solution (1 M, 20 mL) was added via pipette and recording continued until no visual change was evident.

For Figure 8 experiments were conducted at 120 and 360 RPM. Recorded using a Microsoft LifeCam Studio webcam and Windows 11 laptop camera app. For Figures 13 and 14, experiments were conducted at 120 RPM with subsurface and impeller zone addition and the hydrochloric acid was added as fast as possible by squeezing the pipette bulb. Recordings for all figures (8, 13 and 14) were made using an iPhone 14 Pro, with fixed focus.

Iron Chloride Displacement Reactions (Figure 10 – 12)

A 2 L aqueous solution of iron(III) chloride (0.2703 g, 1 eq) was prepared and transferred to the reactor. Stirring was commenced and recording started, iPhone 14 pro. Ammonium thiocyanate aqueous solution (0.2283 g, 1 eq, 20 mL) was added after one minute and recording continued for at least 30 seconds. Experiments were conduct at 200 RPM and 400 RPM, baffled and unbaffled.

Computational Details

Figure 4 - CFD models

Computational Fluid Dynamics Modelling with Ansys R17.1

Ansys Workbench was used to manage all required steps for a model and subsequent postprocessing (geometry, mesh, setup, solution, and results):

Geometry
Reactor geometry was built in the Design modeler, a computer aided design (CAD) software within Ansys for building virtual models of the system of interest.

Using a combination of basic shapes (primitives) and lines, it is simple to create 2D systems. Naturally, 3D systems are more involved, using 3D primitives and more complex shapes from 2D sketches 3D objects can created with the extrude tool.

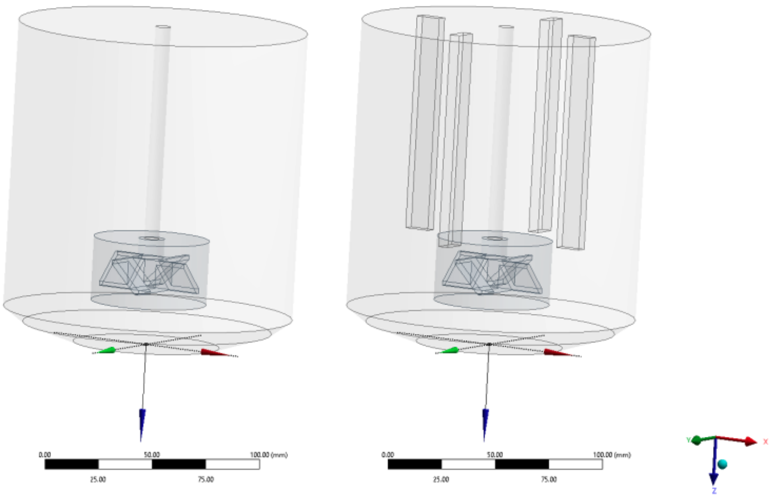

Geometry - Tank Creation (Based on Asynt Specification Sheet)

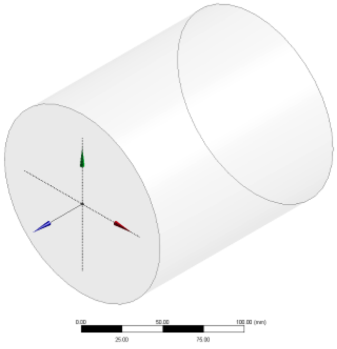

Primitive cylinder
Z length – 150 mm
Radius – 67.5 mm

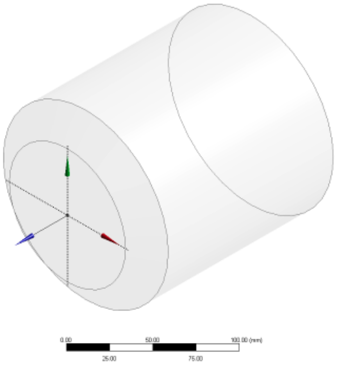

Chamfer
Left – 15 mm
Right – 20 mm

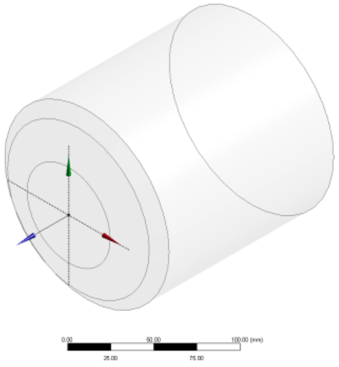

Chamfer
Left – 40 mm
Right – 40 mm

Geometry – Baffle Creation

Primitive Box
Origin X – 35 mm
Origin Y – -2.5mm
Origin Z – -160 mm
X Length – 12 mm
Y Length – 5 mm
Z Length – 110 mm

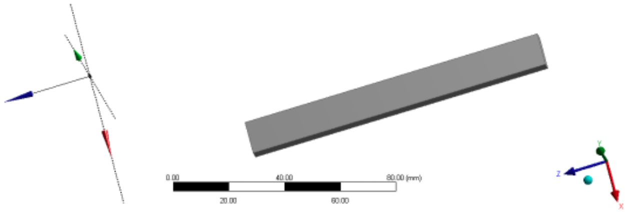

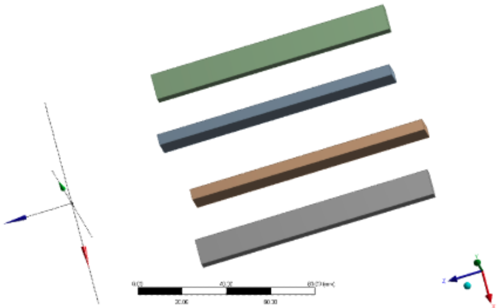

Pattern – Circular

Evenly spaced, three copies

Geometry – Impeller Creation

Primitive Cylinder

Origin Z – -40 mm
Z Length – -150 mm
Radius – 3.5 mm

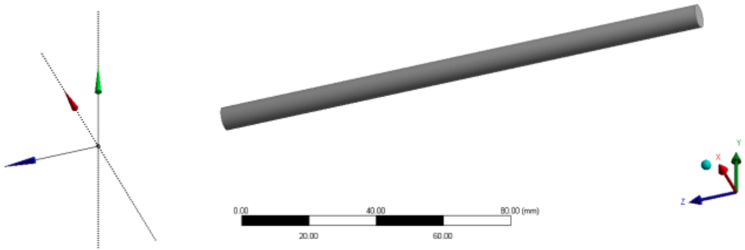

Primitive Cylinder

Origin Z – -50 mm
Z Length – 12 mm
Radius – 6 mm

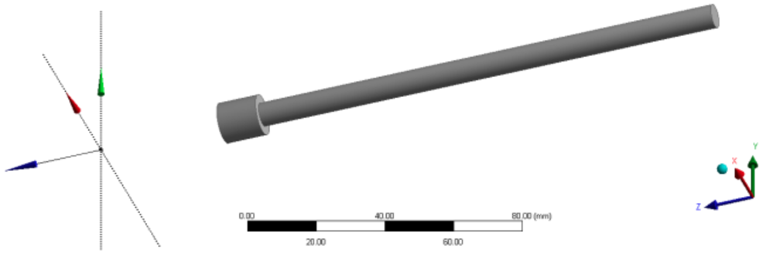

Primitive Box

Origin X – -7.5 mm
Origin Y – -7.5mm
Origin Z – -41 mm
X Length – 30 mm
Y Length – 15 mm
Z Length – 15 mm

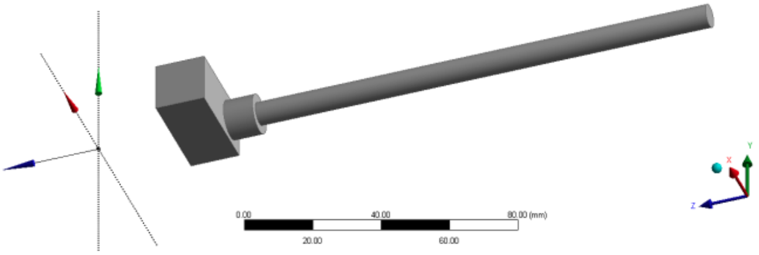

Sketch on the face of the box.

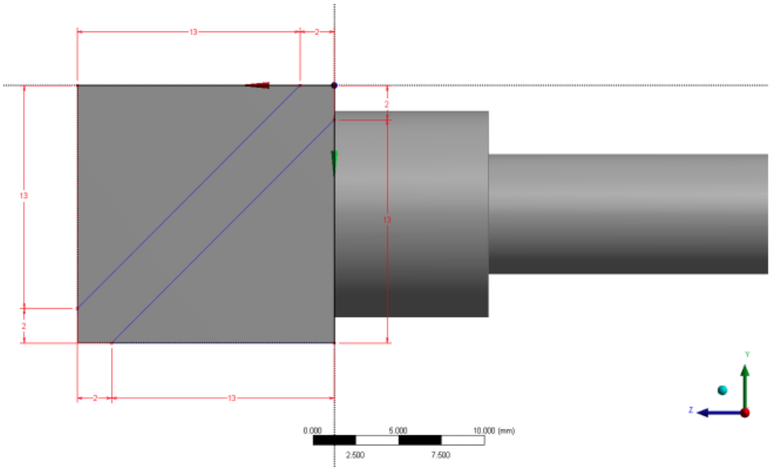

Extrude to cut 15 mm based on the sketched area.

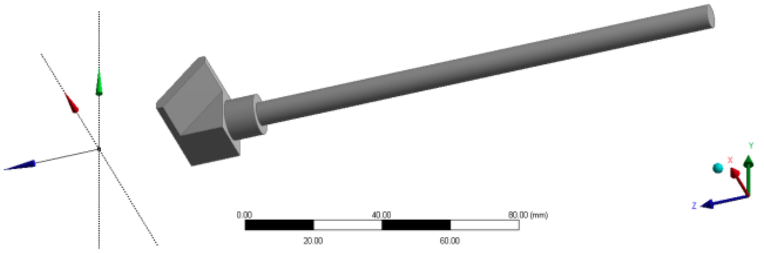

Pattern – Circular

Evenly spaced, three copies.

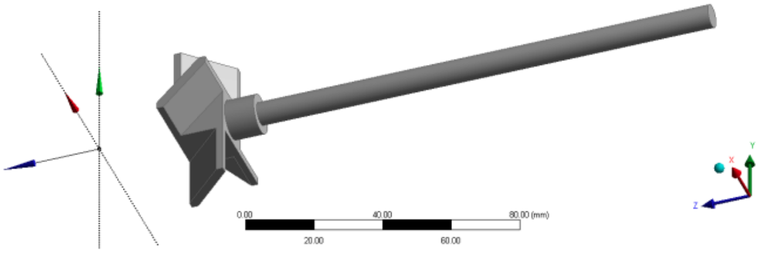

Meshing

To perform calculations on the geometries, a 2- or 3-dimensional grid (or 'mesh') must be generated. It sets the discretization of the domain. Meshing is required for the finite element analysis which Ansys uses to numerically solve the Navier-Stokes differential equations. Typically, the more elements, the more accurate the solution, but this is at the expense of computational time. A good mesh reduces the time until an accurate solution is converged upon. Beyond setting the typical element length Ansys meshing is generally reliable at generating a good mesh. Once a mesh has been generated it is important to check the mesh statistics. In this work, three statistics where focused on: aspect ratio (< 5), skewness (< 0.7), and orthogonal quality (> 0.3):

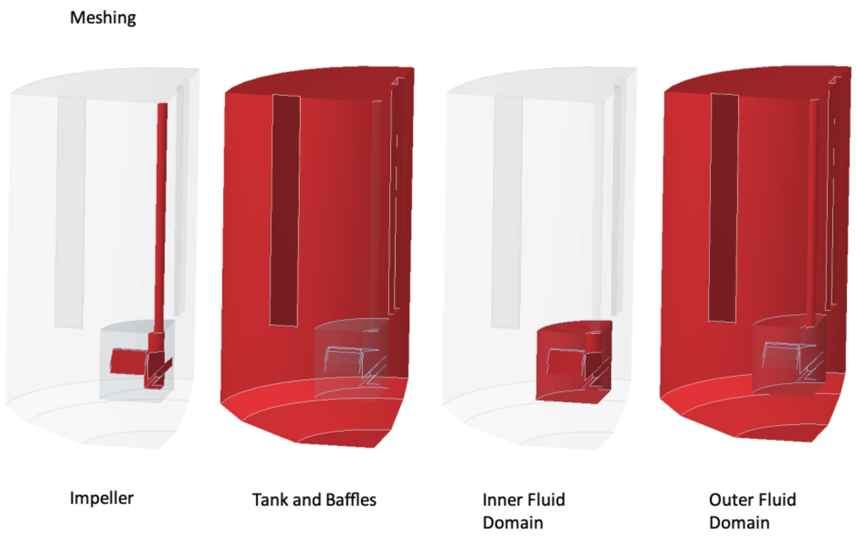

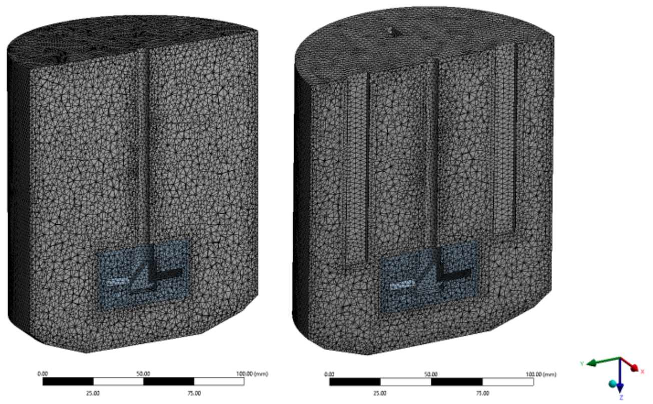

				Aspect Ratio		Skewness		Orthogonal Quality	
Setup	Element size (mm)	Nodes	Elements	Average	Std	Average	Std	Average	Std
Baffled	0.002	115138	594068	1.863	0.465	0.23454	0.12214	0.855	0.084
Unbaffled	0.002	113908	593031	1.853	0.46	0.23018	0.12101	0.857	0.083

Setup

The first step is to change the precision and number of computer cores allocated to the problem. CFD is computational expensive. All simulations used double precision and at least 12 processing cores to reduce runtime:

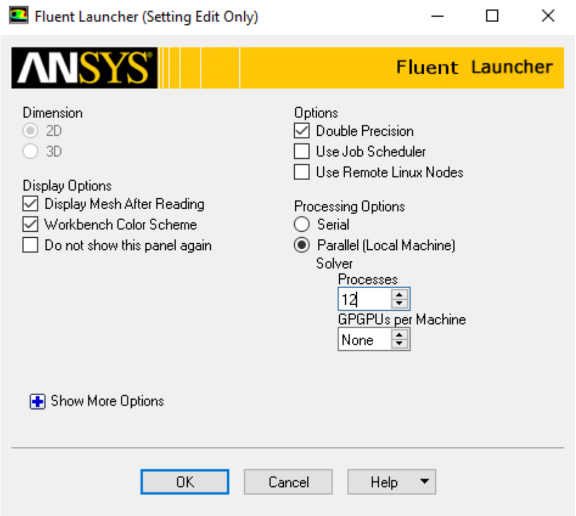

All models used the material properties of water. Two viscosities were used, 0.001003 and 0.0009 kg/m/s:

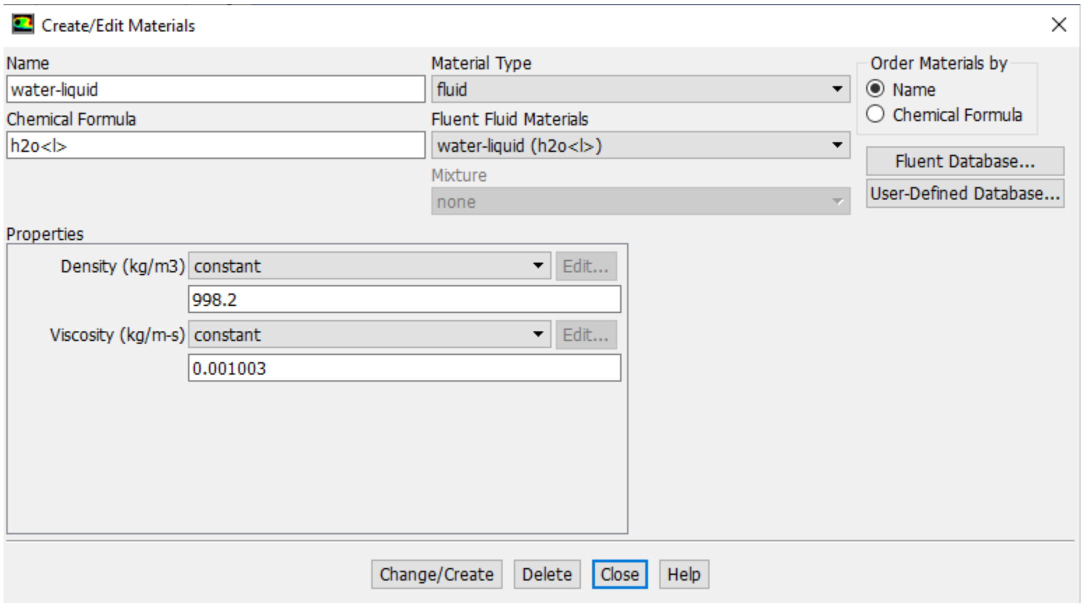

Additional setup details:

Setup

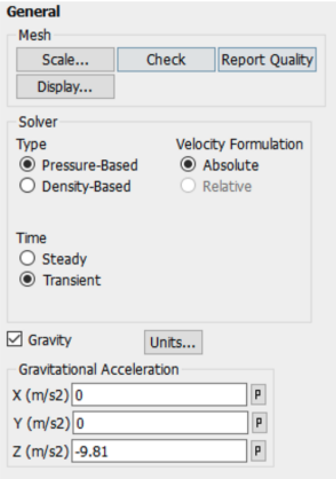

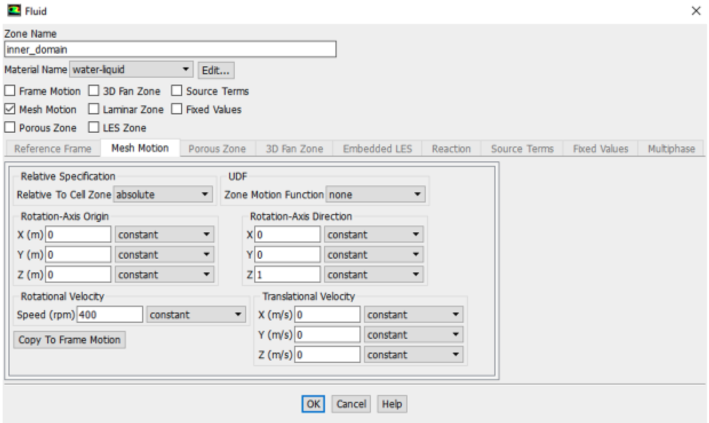

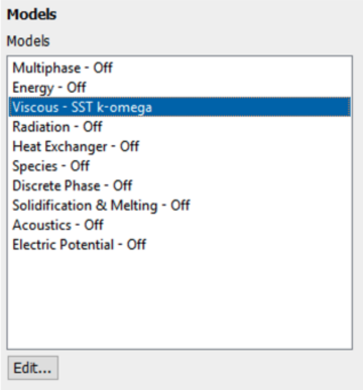

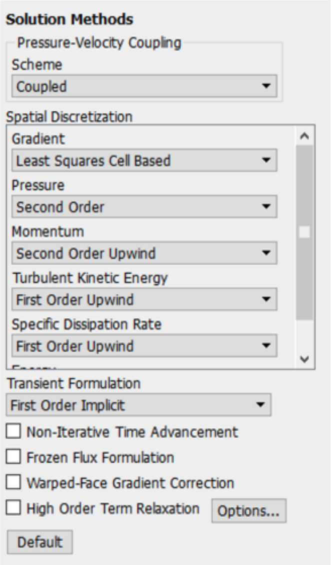

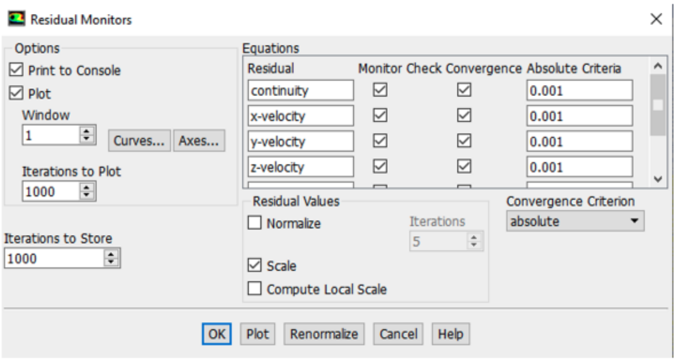

Table 1 - CFD Difference Maps

Table 1 of the manuscript shows CFD difference maps for visualising the velocity changes between any two CFD models, with the same mesh, along the same plane in each model.

First, the variables of interest from the two models were consolidated in one spreadsheet, from Ansys output data labelled "outer domain".

The variables that were used were:

- X [m],
- Y [m],
- Z [m], and
- Velocity in Stn Frame [m s^-1].

The data sets were filtered to include only the coordinates that were within ± 0.001 m along the X-axis.

The reduced data set bisected the reactor creating a YZ plane that matches the YZ velocity contour plot generated during model postprocessing.

The two data sets coordinates were matched, and the velocity difference and unsigned difference calculated.

The mean and median velocity changes were calculated from the velocity difference. The unsigned velocity difference along with the YZ coordinates were used to create a bubble plot to show the magnitude of the change across the plane.

For any data to be displayed on these plots, there had to be a *positive* influence on the velocity on moving from 200 to 400 RPM stirring rate.

All machine-readable spreadsheet files and exemplar Python scripts for processing these data and generating CFD difference plots are available in the zipped folder uploaded as part of the supporting information:

https://figshare.com/articles/dataset/Supporting_Information_zip/26405800

Table 2 - DynoChem Calculations

DynoChem calculations were based on the geometry and material properties used in the CFD calculations. Summary of the calculations shown in the below **Table S1**:

Table S1:DynoChem Mixing Summary

		Version 6.0.5	Version 6.0.5	Version 6.0.5	Version 6.0.5	Version 6.0.5	Version 6.0.5
		14/11/2023 11:46	14/11/2023 11:46	14/11/2023 11:46	14/11/2023 11:49	14/11/2023 11:49	14/11/2023 11:50
**Mixing duty**	Location selected	Any location	Any location	Any location	Any location	Any location	Any location
	Vessel ID	<User-defined Vessel>	<User-defined Vessel>	<User-defined Vessel>	<User-defined Vessel>	<User-defined Vessel>	<User-defined Vessel>
	Maximum volume (L), from database	0.00	0.00	0.00	0.00	0.00	0.00
	Scale factor (compared to Vessel 1)	1.00	1.00	1.00	1.00	1.00	1.00
Liquid	Liquid volume (L)	2.00	2.00	2.00	2.00	2.00	2.00
	Liquid level (mm)	149	149	149	149	149	149
	Liquid mass (kg)	2.01	2.01	2.01	2.01	2.01	2.01
Heat transfer	Total surface area (m2)	0.07	0.07	0.07	0.07	0.07	0.07
**Performance**	Agitator speed, N (rpm)	120	200	400	120	200	400
	Power input per unit mass, e (W/kg)	0.001	0.003	0.019	0.001	0.006	0.048
	Reynolds number, Re (-)	3.97E+03	6.62E+03	1.32E+04	3.97E+03	6.62E+03	1.32E+04
	Vessel flow regime	Transitional	Transitional	Turbulent	Transitional	Turbulent	Turbulent
	Tip speed, V_{tip} (m/s)	0.28	0.47	0.94	0.28	0.47	0.94
	Mixing time (s)	51.84	19.72	9.81	31.60	14.35	7.18

**Physical properties**							
**Liquid**	Liquid density, r_L (kg/m3)	1001.40	1001.40	1001.40	1001.40	1001.40	1001.40
	Liquid dynamic viscosity, m (cP)	1.02	1.02	1.02	1.02	1.02	1.02
**Vessel Geometry**							
**Tank**	Base shape	DIN Torispheri cal	DIN Torispheri cal	DIN Torispheri cal	DIN Torispheri cal	DIN Torispheri cal	DIN Torispheri cal
	Inner diameter, T (mm)	135	135	135	135	135	135
	Total height to tan (mm)	190	190	190	190	190	190
	Maximum volume (L), from database	0.00	0.00	0.00	0.00	0.00	0.00
	Base height (mm)	26.2	26.2	26.2	26.2	26.2	26.2
	Base volume (L)	0.24	0.24	0.24	0.24	0.24	0.24
**Bottom impeller**	Impeller type	4-bladed pitched blade turbine	4-bladed pitched blade turbine	4-bladed pitched blade turbine	4-bladed pitched blade turbine	4-bladed pitched blade turbine	4-bladed pitched blade turbine
	Tip diameter (mm)	45.0	45.0	45.0	45.0	45.0	45.0
	Clearance (mm)	25.0	25.0	25.0	25.0	25.0	25.0
	Projected blade width (mm)	15.0	15.0	15.0	15.0	15.0	15.0
	Impeller Power Number (Po)	0.8	0.8	0.7	1.8	1.8	1.8
	Impeller S or z number (-)	4.4	4.4	4.4	4.4	4.4	4.4
**Impeller 2**	Impeller type	None	None	None	None	None	None
	Tip diameter (mm)	0.0	0.0	0.0	0.0	0.0	0.0
	Clearance (mm)	0.0	0.0	0.0	0.0	0.0	0.0
	Projected blade width (mm)	0.0	0.0	0.0	0.0	0.0	0.0
	Impeller Power Number (Po)	0.0	0.0	0.0	0.0	0.0	0.0
**Impeller 3**	Impeller type	None	None	None	None	None	None
	Tip diameter (mm)	0.0	0.0	0.0	0.0	0.0	0.0
	Clearance (mm)	0.0	0.0	0.0	0.0	0.0	0.0

	Projected blade width (mm)	0.0	0.0	0.0	0.0	0.0	0.0
	Impeller Power Number (Po)	0.0	0.0	0.0	0.0	0.0	0.0
**Impeller 4**	Impeller type	None	None	None	None	None	None
	Tip diameter (mm)	0.0	0.0	0.0	0.0	0.0	0.0
	Clearance (mm)	0.0	0.0	0.0	0.0	0.0	0.0
	Projected blade width (mm)	0.0	0.0	0.0	0.0	0.0	0.0
	Impeller Power Number (Po)	0.0	0.0	0.0	0.0	0.0	0.0
**Baffles**	Degree of baffling (%)	0% baffled	0% baffled	0% baffled	100% baffled	100% baffled	100% baffled

Plateau Analysis

General Method

The methods reported herein were first reported by our group in the following publication:

Chem. Sci., **2023**,*14*, 11872-11880, DOI: https://doi.org/10.1039/D3SC01383A, and supporting information therein.

The underlying principle of the plateau detection method is plateau is a region of little or no change; the 1st order differential will be approximately zero. Employing numerical differentiation, interpreted as calculating the gradient between two adjacent points, noise is amplified when values and associated error are of similar magnitude. This is often the case at a plateau when changes in the y-axis values are small. Data smoothing via rolling averages was employed to minimise the impact of noise. When the rolling average of the gradient is below a defined threshold value, it may be in a plateau region. A plateau region was formally defined if a user-defined number of consecutive points were below the threshold rate-of-change value.

The user defined values are summarised in **Table S2**:

Table S2: User defined values used in the plateau analysis for endpoint times report.

	Rolling Average Number of Points	Minimum Number of Point	Maximum Gradient
200 RPM Baffles	5	10	2
200 RPM No Baffles	5	10	2
400 RPM Baffles	5	10	2
400 RPM No Baffles	5	10	2
Table 4 - Plateau Analysis for Villermaux-Dushman Stirring Rate Study			
40 RPM	9	100	0.25
120 RPM	9	100	0.25
360 RPM	9	100	0.25
Table 5 - Plateau Calculations for FeSCN Reactions (b*)			
200 RPM Baffles	3	3	0.5
200 RPM No Baffles	3	3	0.5
400 RPM Baffles	3	3	0.5
400 RPM No Baffles	3	3	0.5
Table 5 - Plateau Calculations for FeSCN Reactions (Entropy)			
200 RPM Baffles	10	80	0.025
200 RPM No Baffles	10	80	0.025
400 RPM Baffles	10	80	0.025
400 RPM No Baffles	10	80	0.025
